# Pulcherrimin and Beyond: The Multifaceted Role of *Metschnikowia pulcherrima* in Postharvest Disease Management—A Scoping Review

**DOI:** 10.3390/jof12040298

**Published:** 2026-04-21

**Authors:** Juliana Pereira Rodrigues Belas, Caroline Corrêa de Souza Coelho, Leda Maria Fortes Gottschalk, Elisa d’Avila Costa Cavalcanti, Denise Maria Guimarães Freire, Otniel Freitas Silva

**Affiliations:** 1 Food and Nutrition Graduate Program, Federal University of the State of Rio de Janeiro (UNIRIO), Avenida Pasteur 296, Rio de Janeiro 22290-240, RJ, Brazil; rpjulians@gmail.com; 2 Embrapa Roraima, Avenida Brasil, 3911, Boa Vista 69315-292, RR, Brazil; caroline.coelho@embrapa.br; 3 Embrapa Agroindústria de Alimentos, Avenida das Américas 29501, Rio de Janeiro 23020-470, RJ, Brazil; leda.fortes@embrapa.br; 4 Institute of Chemistry, Federal University of Rio de Janeiro, Avenida Athos da Silveira Ramos, Bloco A, 534-A, Cidade Universitária, Rio de Janeiro 21941-909, RJ, Brazil; elisa@iq.ufrj.br (E.d.C.C.); freire@iq.ufrj.br (D.M.G.F.)

**Keywords:** *Metschnikowia pulcherrima*, biocontrol, postharvest, fungal diseases

## Abstract

Postharvest losses of fruits and vegetables are a global problem that directly affect food security, the economy, and the environment. These losses are mainly associated with fungal diseases during storage. Due to the limitations of synthetic fungicides, including the development of resistance and risks to human health, there is growing interest in sustainable disease control strategies. This scoping review analyzes the potential of the yeast *Metschnikowia pulcherrima* as a biocontrol agent for postharvest phytopathogens, based on the scientific literature published between 2014 and 2026. The reviewed studies identify several antagonistic mechanisms, including competition for nutrients and space, the production of organic volatile compounds, hydrolytic enzyme activity, biofilm formation, and the induction of resistance in fruits. In vitro and in vivo assays show that *M. pulcherrima* effectively reduces postharvest disease incidence and severity caused by certain fungi. Furthermore, its synergistic effect when combined with emerging technologies is notable. The results highlight its potential as a sustainable alternative to synthetic fungicides, although further studies are needed for large-scale commercial application.

## 1. Introduction

Postharvest diseases caused by fungi account for a significant portion of production losses, especially when proper management is not implemented during the preharvest and postharvest phases [1].

The postharvest period is a critical stage in the production of foods such as fruits and vegetables, due to the high rates of losses that occur for various reasons, including improper handling, transport, storage, deterioration by microorganisms, and physiological degradation [2]. In 2015, emphasizing the need to prevent food losses and waste, the United Nations adopted the 2030 Agenda for Sustainable Development establishing the reduction in food loss and waste along production and supply chains as one of the targets of the 12th Sustainable Development Goal (SDG) within this Agenda [3,4].

Currently, chemical, physical, and biological treatments are used on fresh fruits and vegetables to prevent food spoilage during the postharvest period. Physical treatments include low temperatures and humidity control, especially during transport and storage, thermotherapy using higher temperatures to treat certain fruits, and controlled or modified atmospheres to reduce water loss [1].

Chemical treatments, such as the application of synthetic fungicides, help control microbial distribution in fruits and vegetables. However, these treatments result in high residue accumulation in food, environmental contamination, and often cause resistance in certain microorganisms, requiring higher chemical doses to remain effective [1,5].

The search for alternatives to synthetic fungicides has generated interest in developing sustainable methods for controlling fungal diseases, such as treatments with products of biological origin, because a significant reduction in the use of chemical products and their residues in the environment, including fungicides, is recognized worldwide as a fundamental premise for achieving sustainable development [3,6].

Biological control is an emerging technique for managing diseases and pests in agriculture and is already widely used worldwide. This method involves the direct or indirect actions of beneficial microorganisms on phytopathogenic microorganisms through complex mechanisms and modes of action, such as competition for space and nutrients, antibiosis, mycoparasitism, and the induction of resistance. It is recognized as a safe, sustainable, and promising approach [7]. In this context, using yeasts as biocontrol agents can support sustainable agricultural practices and reduce postharvest losses [5]. Yeasts from the genera *Candida*, *Cryptococcus*, *Pichia*, *Metschnikowia*, *Rhodosporidium*, *Wickerhamomyces*, *Hanseniaspora*, and *Saccharomyces*, among others, have been widely studied for biocontrol in recent years [6,8].

Antagonistic yeasts are non-filamentous, unicellular fungi that can inhibit the growth of other microorganisms through mechanisms such as competition for space or nutrients and the induction of systemic resistance in the host [7]. According to Oztekin and Karbancioglu-Guler [5], these yeasts are classified as GRAS (generally recognized as safe) by the US Food and Drug Administration (USFDA) because they do not produce mycotoxins, antibiotics, or spores that could cause allergies. Additionally, these biocontrol agents have stable genetics, reproduce on low-cost substrates without the need for special nutrients, are resistant to adverse environmental conditions, can colonize dry surfaces for long periods, are effective against a wide range of fruit pathogens, and have some resistance to pesticides [5,9,10].

According to He et al. [11], postharvest biological control is a system involving antagonistic yeasts, phytopathogens, and fruits or vegetables as hosts, with their interactions being strongly influenced by environmental conditions. Therefore, selecting appropriate yeasts, understanding the behavior of each group (antagonist, pathogen, and host), and optimizing environmental conditions determine the effectiveness of biological control.

Non-*Saccharomyces* and unconventional yeasts, such as *M.* *pulcherrima*, have attracted attention for their diverse biocontrol properties and versatility, including competition for space and nutrients—mainly through the production of pulcherrimin—biofilm formation, the production of hydrolytic enzymes, volatile organic compounds (VOCs), and mycocins, and the induction of systemic resistance [12]. Studies also show promising results in controlling phytopathogenic microorganisms, such as fungi of the genera *Penicillium*, *Botrytis*, *Colletotrichum*, *Alternaria*, *Aspergillus*, and others, which can cause significant postharvest diseases such as rot and mold [10,13,14,15,16].

So far, no specific scoping review on the use of *M. pulcherrima* as a biocontrol agent in postharvest management has been identified in the scientific literature reviewed. Therefore, the objective of this review is to highlight the various modes of biological control action of this yeast and its combined use with emerging technologies, which have demonstrated a positive synergistic effect and can serve as an alternative to traditional synthetic fungicides, benefiting the environment. To achieve this, a search was conducted for original articles published in journals indexed in various scientific databases from 2014 to 2026 to select studies relevant to the topic.

## 2. Materials and Methods

### 2.1. Design and Development of Research Questions

This study was an exploratory analysis using a scoping review of the literature on the role of the yeast *M. pulcherrima* as a biocontrol agent for postharvest diseases and phytopathogens. The review was planned and conducted according to the Joanna Briggs Institute (JBI) manual and followed the guidelines of the Preferred Reporting Items for Systematic Reviews and Meta-Analyses extension for scoping reviews (PRISMA-SCR) [17,18].

To ensure methodological rigor in the development of this review, a protocol was registered in the Open Science Framework (OSF) and is available for public consultation at https://doi.org/10.17605/OSF.IO/3HCRS. The study structure was based on the PCC (Population, Concept, and Context) model [18]. The model was defined as follows:

P = population (*M. pulcherrima* yeast); C = concept (antagonistic action in disease control, biocontrol); and C = context (postharvest period).

The study was based on the following questions: (1) What is the antagonistic potential of *M. pulcherrima* yeast in controlling postharvest diseases in fresh foods?; (2) What are the possible modes of biocontrol action of *M. pulcherrima* against phytopathogens and postharvest diseases?; (3) Are there records of the combined use of *M. pulcherrima* with other postharvest treatments?; and (4) Are there currently any products on the market formulated with *M. pulcherrima* for the protection of food against postharvest diseases?

### 2.2. Definition of the Search Strategy

Journal searches were conducted at two different times, in February 2025 and March 2026, using the Scopus, ScienceDirect, Web of Science, PubMed, EBSCO, and CAB Abstracts databases. To increase the sensitivity of the study search, two separate sets of search terms were defined and combined using Boolean operators. Searches were performed in all six databases, limiting the search period from January 2014 to March 2026, selecting the option to search by title, abstract, and keywords whenever available. The following descriptors were used:

Query string 1: (“Metschnikowia pulcherrima” OR “M. pulcherrima” OR “Candida pulcherrima”) AND (“postharvest” OR “post harvest” OR “post-harvest”)
Query string 2: (“Metschnikowia pulcherrima” OR “M. pulcherrima” OR “Candida pulcherrima”) AND (“antagonistic yeast” OR biocontrol OR bioprotection OR biopreservation)

The total number of records from these searches, for both sets of terms, was combined to determine the total number of files found. These files included research articles, review articles, conference papers, book chapters, dissertations, and patents. The complete search strategies for all databases are available at the link provided in Section 2.1.

### 2.3. Selection and Screening of Primary Studies

The results of the database searches were exported, when possible, as .RIS or CSV files to Zotero software (v. 7.0.32) for document management. Duplicate files were removed initially. The remaining files were analyzed by two independent reviewers, who assessed article eligibility by reading the titles and abstracts of each study. Works selected in this first screening and then had their full texts reviewed. In both selection stages, the following inclusion and exclusion criteria were applied:

(i) Inclusion criteria: research articles; works published between January 2014 and March 2026; articles that use *M. pulcherrima* as a postharvest biocontrol agent; and postharvest treatments combined with *M. pulcherrima*. (ii) Exclusion criteria: review articles; book chapters; full text not available in English; articles that do not mention *M. pulcherrima* as a biocontrol yeast; articles not related to the postharvest period; and studies with wine production as the main theme. The reviewers resolved disagreements about study inclusion during their meeting.

### 2.4. Data Extraction and Synthesis

Sixty-one articles that met the defined inclusion criteria were selected. A spreadsheet containing data extracted from the studies was created and distributed to the authors for independent completion.

The data defined for extraction included article title, authors, year of publication, journal, study location, authors’ keywords, *M. pulcherrima* strains used in the studies, origin of the *M. pulcherrima* strain isolations, culture medium and parameters used for *M. pulcherrima* cell multiplication, phytopathogenic microorganisms studied, biocontrol modes of action of *M. pulcherrima* tested in each study, foods used for *in vivo* tests, postharvest treatment methods combined with the use of *M. pulcherrima*, and the main results obtained. The tables were reviewed interactively as needed during the screening of each included article. Any discrepancies were resolved by an independent reviewer through a new review of the study.

### 2.5. Data Analysis

The articles selected for data extraction were analyzed from an .xlsx file using the Biblioshiny application in the Bibliometrix (v.3.2.1) package in RStudio software (v. 2023.06.2). Bibliographic data were processed by collecting information from each article to analyze the annual output, output by country, the journals where the articles were published, and keywords co-occurrence. Graphs, tables, and figures were created using the Biblioshiny application and Microsoft Excel (v. 2603), and the keywords co-occurrence map was created with VOSviewer software (v. 1.6.20).

To organize and simplify the information from the bibliometric analysis, only keywords that appeared at least four times were included in the discussion of the results.

### 2.6. Protocol and Registration

The protocol for this scoping review was registered in the Open Science Framework (OSF) and is available at https://doi.org/10.17605/OSF.IO/3HCRS (created on 18 March 2026), and a Supplementary Material is also available at this link.

## 3. Results and Discussion

### 3.1. Bibliometrics from the Last 12 Years (2014–2026) on the Use of M. pulcherrima as a Postharvest Biocontrol Agent

**Figure 1:**
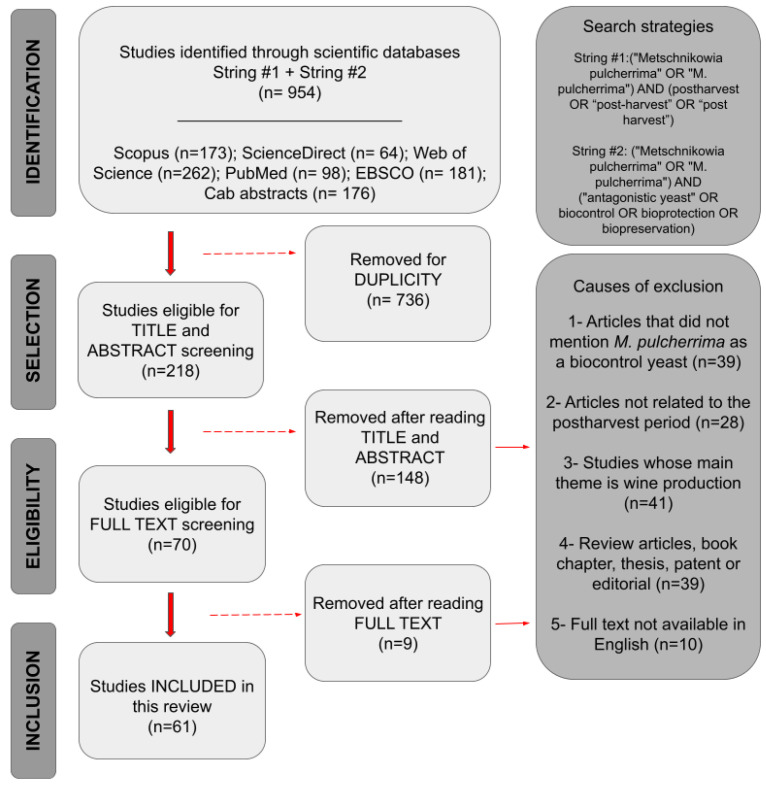
PRISMA flowchart illustrating the study selection process for this scoping review.

Using a defined search strategy, 954 files were retrieved from the selected databases, of which 70 research articles mentioned the use of *M. pulcherrima* as a postharvest biocontrol agent. During the full-text reading phase, nine studies were excluded: three were not available for download, five were in languages other than English, and one was not about postharvest. After this screening, 61 articles were included in the review for the data extraction phase, as shown in the flowchart in Figure 1.

**Figure 2:**
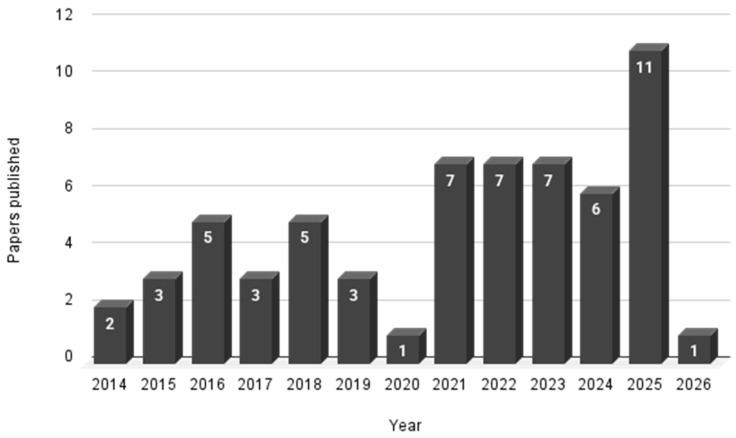
Number of articles selected in the research by year (2014–2026).

According to Figure 2, between 2014 and 2020 the number of publications did not exceed five per year. From 2021 onward, there was a significant increase in articles related to the study of *M. pulcherrima* as a potential biocontrol agent, especially compared to 2020, when there was a sharp drop in publications (*n* = 1). In subsequent years, the number of published articles increased, notably in 2025, with 11 articles. In the remaining years, at least six articles were published per year from 2022 to 2025.

The increase in publications cannot be attributed to a single factor but results from several factors observed over the years, such as advances in genetic sequencing of microorganisms, increased research interest in non-*Saccharomyces* yeasts, a greater global focus on sustainability-related topics, and regulatory pressures to reduce the use of synthetic agrochemicals.

Of the 61 articles selected for this review, the potential of *M. pulcherrima* as a biocontrol agent was tested in various foods, including as apples [19,20,21,22], oranges [23], mangoes [24,25], strawberries [16], grapes [26,27,28,29], and potatoes [30], among others. Additionally, some selected articles reported the effectiveness of *M. pulcherrima* biocontrol against the development of postharvest phytopathogenic fungi that cause rot, such as *Alternaria alternata*, *Botrytis cinerea*, *Penicillium digitatum*, *Penicillium expansum*, *Colletotrichum gloeosporioides*, and *Monilinia fructicola*, among others [24,29,31,32].

Among the countries with the highest number of publications, Italy (11), China (16), and Spain (10) stand out. The remaining works are published mainly in other European countries. Additionally, research partnerships are observed between Ireland, Switzerland and United Kingdom [33], Malaysia and Italy [29], South Africa and Italy [34], France and Italy [31], and Spain and France [35,36].

**Figure 3:**
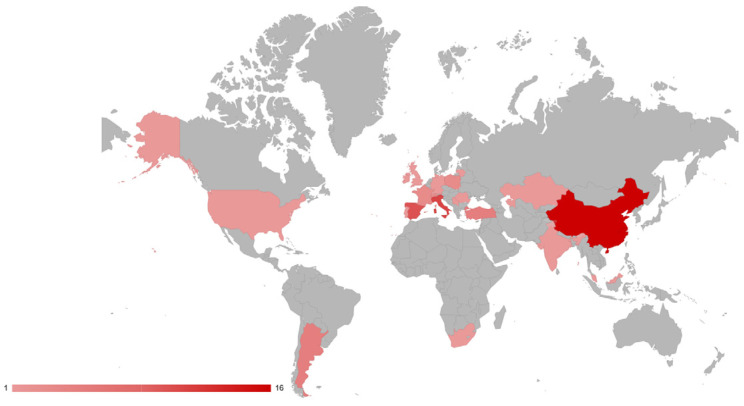
Geographic distribution by country of the scientific output of articles included in the scoping review.

In South America, Argentina stands out with five published studies on *M. pulcherrima*, making it the only country in Latin America to publish these studies internationally (Figure 3). The selected Argentine studies report the use of *M. pulcherrima* for the biocontrol of diseases caused by fungi such as *A. alternata* and *P. expansum* in table and wine grapes [4,26,27,28,37]. This is likely related to the significant role of grapes in the local economy, as they are the second most produced fruit in the country. Additionally, Argentina ranks fifth among the world’s top wine-producing countries, with approximately 10.9 million hectoliters produced that year [38,39].

The analysis of articles published by country is based on the absolute number of publications, which may affect comparisons between countries. Therefore, the results should be interpreted cautiously.

Table 1 lists various international scientific journals that publish research on *M. pulcherrima* during the postharvest period. A total of 36 scientific journals have published articles on the topic covered in this review, underscoring its importance to the scientific community. Data analysis showed that the *International Journal of Food Microbiology* published the most articles on this topic (eight articles), supporting the relevance of studies on biological control using living organisms as biocontrol agents for food safety and quality, as this journal focuses on these areas. The journals *Food Microbiology*, *Biological Control*, and *Microorganisms* each published five articles on the topic of this review. Overall, these journals publish articles directly or indirectly related to microorganisms and their interactions with biological systems, food, and ecosystems.

The two studies published in the journal *Toxins* examine the use of *M. pulcherrima* as a biocontrol agent against postharvest phytopathogenic fungi such as *Aspergillus flavus* and *P. expansum*, which produce mycotoxins like AFB_1_ and patulin. The journal aims to disseminate research on biotoxins, toxinology, and toxicology, including food toxicology.

**Figure 4:**
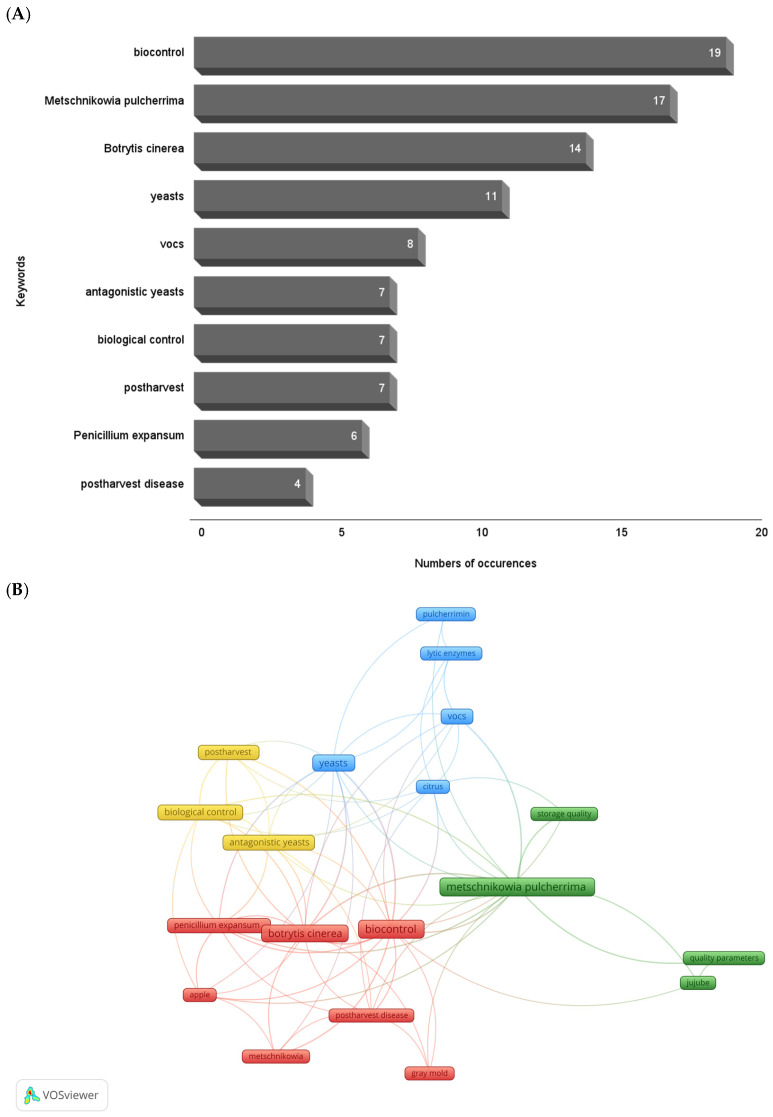
Main author keywords identified in the selected articles from the journal search (**A**) number of occurrences of the same keywords and (**B**) co-occurrence map of keywords.

Figure 4A shows the keywords used by the authors in the 61 selected articles that appear at least three times. The most frequently mentioned words relate to the genus and species of the studied yeast, “*Metschnikowia pulcherrima*”, and its use in “biocontrol” studies. Other prominent keywords are associated with biocontrol mechanisms, such as the production of “VOCs” and “pulcherrimin”, a pigment formed by a chelated complex between ferric iron (Fe^3+^) and pulcherriminic acid produced by *M. pulcherrima*. Some keywords also refer to the intended application of these yeasts, such as “postharvest” and “postharvest disease”. The names of fungi that cause postharvest diseases, such as *P. expansum* and *B. cinerea*, are also frequently cited as keywords, likely due to the significant economic losses caused by these diseases.

The keywords used by authors in articles represent the central content of the study and the focus of the topics covered, while the co-occurrence of keywords across different articles reflects the conceptual and thematic relationships between the main topics addressed in research or within a set of publications [75].

Figure 4B shows the keyword co-occurrence analysis performed using VOSviewer software (v. 1.6.20). The network analysis identified four thematic groups (clusters), each represented by a different color (red, blue, green, and yellow), which structure the research field on the use of *M. pulcherrima* yeasts as a biocontrol agent for postharvest food applications. VOSviewer typically organizes keyword clusters around frequent research themes, with strong internal links and more specific connections to other groups. When nodes are close together and connections are strong, this indicates that the terms co-occur more frequently and have greater conceptual integration. Cluster 1 (red), composed of seven keywords (“biocontrol”, “apple”, “*Botrytis cinerea*”, “*Penicillium expansum*”, “postharvest disease”, “*Metschnikowia*”, and “gray mold”), focuses on studies using yeasts as biocontrol agents against major postharvest pathogens such as the fungi *B. cinerea* and *P. expansum*, which cause significant losses in fruits like apples. This cluster may represent the practice of biological control strategies. Cluster 2 (blue) groups five keywords related to the mechanisms of action likely used by *M. pulcherrima* in its biocontrol activity, including “VOCs”, “pulcherrimin”, “lytic enzymes”, “citrus” and “yeasts”. This suggests a line of research focused on understanding the chemical and metabolic mechanisms involved in microbial antagonism. The inclusion of “citrus” in this cluster highlights the frequent use of citrus fruits in tests involving these mechanisms. Cluster 3 (green), composed of four keywords (“quality parameters”, “jujube”, “storage quality” and “*Metschnikowia pulcherrima*”), suggests an interest in evaluating the effects of *M. pulcherrima* application on fruit quality during storage. The mention of jujube fruit may indicate a focus on studying these quality parameters in this fruit. Cluster 4 is the smallest cluster, colored yellow, and includes the words “postharvest”, “biological control” and “antagonistic yeast”. It is closely related with both the red and blue clusters, and possibly highlights research on biocontrol strategies that employ yeasts as antagonistic agents to control diseases during postharvest period. The co-occurence map shows a trend in studies aimed at elucidating the biocontrol mechanisms of *M. pulcherrima*, focusing on expanding knowledge of specific pathogen–host interactions and exploring the practical applications of biological control in postharvest management of various fruits.

### 3.2. Metschnikowia pulcherrima

Yeasts are microorganisms that have been used for thousands of years as fermentation agents in the production of foods such as bread, wine, yogurt, and cheese [7,76]. Non-*Saccharomyces* or non-traditional yeasts, such as those of the *Metschnikowia* genus, have gained prominence in recent years due to their biological characteristics applicable in the agricultural, oenological, and cosmetic industries [76]. Characteristics such as their abundant presence in natural substrates, easy isolation, simple nutritional requirements, low cost, colonization capacity, survival on dry surfaces for long periods, rapid growth, absence of allergenic spore or mycotoxin production, and phenotypes adapted to survival on the surface of fruits and vegetables make yeasts suitable antagonists for use as biocontrol agents [44,77,78].

The species *M. pulcherrima* was first identified by this name in 1968 by Pitt and Miller [79], but there are reports of its existence under other names, such as *Torula pulcherrima* Lindner or *Candida pulcherrima*, now referred to as its asexual reproductive form, dating back to the early 1900s [79,80,81]. After an extensive review of works published between 1901 and 1967 on the morphological characteristics of the yeast *M. pulcherrima*, an article was published in 1968 reporting taxonomic and ecological discoveries about this species [79].

The Latin word *pulcherrima* means “the most beautiful”, referring to the uniform and spherical shape of the yeast cells when viewed under a microscope, without associating it with the iron-chelated complexes, which are red in color [33,80].

**Figure 5:**
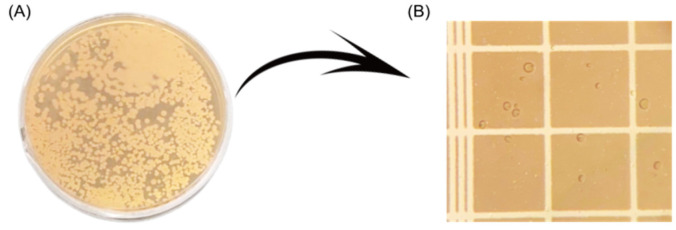
Observation of *M. pulcherrima* DSM 70336 cells cultivated in malt extract culture medium (**A**), and photographic section of the Neubauer chamber (**B**).

According to Miller and Phaff [81], when the yeast *M. pulcherrima* is cultivated in 5% malt extract medium, growth observed after 3 days at 25 °C shows single globose to ellipsoidal vegetative cells, measuring approximately 2.5–6 × 4–10 µm, which reproduce by multilateral budding, [81], as shown in Figure 5. Vegetative spores (chlamydospores) appear only after about 30 days from the start of growth. These highly resistant spores are globose or subglobose in shape, measuring 4 × 7–11 µm, and they generally contain a single lipid globule. A thin ring and abundant sediment are usually present in these spores, which lack a pellicle. When this yeast is cultivated in 10% malt extract medium for one month, the growth appears cream-colored in the absence of the pulcherrimin pigment, or reddish-brown in sectors in the presence of the pigment, which generally also diffuses into the culture medium. The colony surface is smooth, shiny, and, depending on the strain, sparsely papillate.

According to Sipiczki et al. [12], yeasts of the genus *Metschnikowia* can be easily found and isolated from natural substrates, fermented drinks, and processed food products. However, identifying their taxonomic position within the phylogenetic branch is difficult due to the frequent occurrence of ambiguous nucleotides (di- or polymorphic) in the D1/D2 and ITS barcode sequences, which are commonly used for taxonomic identification of yeast strains. This difficulty is further compounded by the presence of these ambiguous nucleotides in the database sequences of reference strains for certain species.

For a long time, yeasts of the genus *Metschnikowia*, due to their similar phylogenetic relationships, were classified as belonging to the *pulcherrima* clade, which included comprised eight species: *M. andauensis*, *M. fructicola*, *M. leonuri*, *M. pulcherrima*, *M. rubicola*, *M. shanxiensis*, *M. sinensis*, and *M. ziziphicola*, as well as two groups of strains with names considered invalid in Mycobank: *M. citriensis* and *M. persimmonesis* [12,82]. However, recent research by Sipiczki and Czentye [83], based on the analysis of primary and secondary barcode sequences of type cultures and their mating tests, revealed that the species in this clade cannot be separated by barcode gaps or reproductive barriers and must be united into a single species, under the older name *M. pulcherrima*.

*M. pulcherrima* yeasts exhibit plastic morphology, alternating between a unicellular state and forming pseudohyphae. The formation of these structures aids yeasts’ attachment to solid substrates by enabling invasive growth, which facilitates colonization and improving adhesion. Additionally, the pseudomycelium formed by pseudohyphae can create biofilms in lesions, further supporting colonization and preventing pathogen entry [84].

Due to high intragenomic and intergenomic variability, as well as the great similarity in morphological characteristics such as carbon consumption, growth intensity, ascus and chlamydospore formation, penetration into solid media through pseudohyphae, and pulcherrim production, differentiation between species is very difficult [12,84]. This hinders the patenting of biotechnological strains of *M. pulcherrima* because of uncertainty in sequencing these microorganisms. Unifying the nomenclature of these different species will likely require screening these yeasts based on their performance and conducting in-depth genomic characterization to differentiate between strains and develop new biotechnological products, considering their microbial activity, pulcherrimin production, and metabolic profile [84].

*M. pulcherrima* has a plastic genome, with its size varying depending on the scientific strain. The APC 1.2 strain has a genome size of 15.88 Mbp, while among the strains NRRL Y-7111, CBS 10357, NRRL Y-48695, and NRRL Y-48712, the size ranges from 24.15 Mbp to 30.85 Mbp [33,85]. Currently, only one strain of *M. pulcherrima* has its chromosomes identified and annotated in the NIH genome database (GenBank^®^): *Metschnikowia aff. pulcherrima* APC 1.2, which is organized into seven nuclear chromosomes, numbered I to VII from largest to smallest [86].

The main biocontrol mechanism of the species, pulcherrimin formation, is regulated by the SNF2 gene, a transcriptional regulator essential for antifungal activity and pulcherriminic acid production. The biosynthesis of pulcherriminic acid, and thus pulcherrimin, depends on specific transport genes in the PUL cluster (PUL1, PUL2, PUL3, and PUL4), which enable the chelation of iron ions outside the yeast cell, making this nutrient unavailable to pathogens and inhibiting their growth [33].

As previously mentioned, due to their favorable characteristics as biocontrol agents, these yeasts can be used as antagonistic microorganisms in postharvest applications and may serve as substitutes for traditional synthetic fungicides. For this reason, they are included in commercial biopesticide formulations [20,47,62,78].

*M. pulcherrima*, in addition to meeting the requirements to be considered a biocontrol agent, was also shown to be a highly competitive yeast compared to other yeasts in research by Gross et al. [87]. The authors demonstrated this by cultivating cells of *M. pulcherrima* (strain APC 1.2) and *Hanseniaspora* sp. (APC 12.1) using YNB-melezitose as a carbon source. After 7 days, although the yeasts started with equal cell numbers, *M. pulcherrima* accounted for more than 90% of the cells in BD medium and 60% in YNB-melezitose medium, indicating strong interspecific competition.

According to Parafati et al. [14], isolating and screening antagonistic microorganisms, as well as identifying and understanding their mechanisms of action, are essential for finding efficient biological control agents. Different strains of the yeast *M. pulcherrima* are commonly found on the surfaces of fruits, flowers, leaves, and insects, as well as in juices and wines, since they are part of the natural microbiota, as shown in Table 2.

Some studies show that the origin of *M. pulcherrima* yeast isolates can significantly affect their efficiency as biocontrol agents, and that the effectiveness of biocontrol may be linked to their ability to establish themselves in the host before pathogen contamination and to their speed of colonizing host tissues [31,33,61,62,88].

Hilber-Bodmer et al. [60], showed that in in vitro competition tests between yeasts with strong antagonistic action, those isolated from orchard soil were more antagonistic and had more diverse metabolism than yeasts isolated from apple flowers. However, the authors emphasize that the antagonistic activity of yeasts is also an inherent property of the isolates and their individual characteristics.

Studies by Marsico et al. [31] state that the development of biological control agents (BCAs) is intrinsically linked to the isolation of yeasts directly from their natural environments, as they compete with the plant pathogens present in those environments, indicating a probability of obtaining efficient BCAs. In this study, four of the five strains of *M. pulcherrima* isolated from grape bunches that showed resistance to gray mold exhibited high antagonistic activity in vitro against the fungus *B. cinerea*, the causal agent of gray mold. This information partially diverges from what Steglinska et al. [30] reported in their study, where five strains of *M. pulcherrima* isolated from apple, raspberry, and strawberry fruits and from strawberry flowers showed antagonistic activity, in in vitro tests, against fungi that cause diseases in potatoes.

Janakiev et al. [49] demonstrated that two strains of *M. pulcherrima* (V2 and V7), isolated from the phyllosphere of “Williams”-variety pear trees (leaves and fruits), had an antagonistic effect on several phytopathogenic fungi of the same species, including *Monilinia laxa*, *B. cinerea*, *Alternaria tenuissima*, *Cladosporium cladosporioides*, *Fusarium proliferatum/fujikuroi*, *Fusarium verticillioides*, *Fusarium sporotrichioides*, *Fusarium solani*, *Fusarium oxysporum*, and *Fusarium incarnatum*, all isolated from these samples. The *M. pulcherrima* strains inhibited the radial growth of these fungi by 21.6 to 64.9%, reinforcing the importance of considering tri-trophic interactions in biocontrol (yeast–fungus–host).

Different culture media and cultivation conditions can be used for the growth of *M. pulcherrima*, demonstrating a wide variety of formulations that support the growth and development of these microorganisms. This reinforces the idea that they can grow on low-cost substrates without special nutrients [78]. Various carbon sources, such as monosaccharides and disaccharides, can be used for the growth and development of *M. pulcherrima* [24]. The most common carbon sources used are glucose and dextrose, as shown in Table 2.

Competition for nutrients is a well-known biocontrol mode of action in yeasts of the genus *Metschnikowia*. In addition to competing for iron ions in the culture medium, these yeasts also compete for carbon sources available in the medium, due to their dependence on this nutrient for development, as do most filamentous fungi that cause postharvest diseases [24,61]. Knowledge of the optimal sources of these nutrients is important for successful biocontrol, as they ensure full yeast growth [61].

Tian et al. [24] created artificial wounds in mango fruits and supplemented them with different carbon sources (sucrose, fructose, or glucose at 2% *w*/*v*). They then inoculated these wounds with *M. pulcherrima* and the fungus *C. gloeosporioides*, which causes rot symptoms in several fruits. The fruits were stored at 15 °C and 25 °C. After 10 days, fruits supplemented with glucose showed larger lesion diameters and disease development that was 134.28% (15 °C) and 127.73% (25 °C) greater than the non-supplemented control. When fructose was added at 25 °C, the lesion diameter was 6.5 mm larger than in the non-supplemented treatment. The addition of sucrose did not result in significant differences in lesion diameter or disease development. According to the authors, these results demonstrate that *M. pulcherrima* is capable of competing for nutrients, as fruits without added carbon sources showed much lower disease symptom development, presumably because the yeast competed for the nutrients present in the fruit and limited the development of the fungus.

In YT microplates, Wang and colleagues [61] observed the consumption of glucose, sucrose, and D-xylose by the yeasts *M. pulcherrima* (P01A016 and P01C004). In addition, the two strains consumed D-gluconic acid, glycerol, L-malic acid, maltitol, D-mannitol, and xylitol as carbon sources, either for energy production (oxidation) or for incorporation into the cell (assimilation).

Over the years, some authors have studied the influence of acidifying the pH of the culture medium on the development and antagonistic action of *M. pulcherrima* and have observed that, in general, the antagonistic activity of these yeasts increases their potential for mycelial inhibition in phytopathogenic fungi when exposed to culture media with a more acidic pH (4.5) [10,14,44].

Parafati et al. [10,14] tested the antagonistic activity of several strains of *M. pulcherrima* in culture media with different pH values (6.0 and 4.5). The authors observed that the *M. pulcherrima* strains caused greater inhibition of *P. digitatum*, *Penicillium italicum*, and *B. cinerea* mycelial growth on plates with pH 4.5 than on plates with pH 6.0.

Storing certain foods, such as fresh fruits and vegetables, at temperatures below 10 °C is a widely used preservation technique that extends their shelf life by reducing physiological activity, maintaining physicochemical characteristics, and limiting microorganism growth [2]. After harvest, using low temperatures is a traditional method applied to some foods, often combined with other disease prevention techniques such as fungicides, thermotherapy, and wax applications [89].

According to Parafati et al. [14], achieving parity between the conditions that favor the antagonistic microorganism and those that favor the phytopathogen is important for successful biocontrol. Given the history of limited microorganism development at low temperatures, some authors have investigated the behavior of certain yeasts with biological control potential when exposed to the refrigerated storage temperatures of fresh fruits, as well as how environmental conditions can affect the mechanisms of action of these antagonists [28].

Piano et al. [90] investigated the effects of different storage temperatures on apples artificially inoculated with *B. cinerea* (10^5^ CFU·mL^−1^), and treated with two strains of *M. pulcherrima* (2.33 and 4.4) cell solutions (10^8^ cells·mL^−1^). The fruits were stored either at 22 °C for one week or at 4 °C for 14 days, followed by storage at 22 °C for 5–7 days. The apples’ treatment response showed that at 4 °C both strains completely inhibited the development of *B. cinerea*, while in fruits stored at 22 °C, yeast 2.33 reduced lesion size by 94%, while yeast 4.4 reduced lesion size by 88%, compared to control fruits.

In the study by Rodriguez Assaf and collaborators [28], three strains of *M. pulcherrima* (Mp22, Mp36, and Mp43) reduced the incidence of blue mold caused by various strains of *P. expansum*, achieving average reductions in wound incidence between 6.67 ± 5.77% and 50 ± 10%, and average lesion diameters between 0 and 5.85 ± 0.22 mm in grape berries stored at 0 ± 1 °C.

Mango fruits treated with *M. pulcherrima* cell solutions and stored for 30 days in a cold room at 15 °C showed delayed ripening and greater pulp firmness compared to control fruits treated with distilled water. Yeast treatment also inhibited changes in soluble solids content, total acidity, and vitamin C content, prolonging fruit quality during storage [24].

Yeasts of the genus *Metschnikowia* are generally known to secrete pulcherriminic acid, which sequesters iron ions (Fe^3+^) from their growth substrate and forms a complex called pulcherrimin [12]. This competition for iron ions reduces the availability of this nutrient for pathogen assimilation, thereby restricting pathogen development. However, strains of *M. pulcherrima*, besides competing for nutrients as a biocontrol mechanism, may also produce extracellular hydrolytic enzymes, VOCs with antifungal activity, and toxins with killer activity, form a protective biofilm, and induce resistance in fruits [14,21,30,32].

### 3.3. Use of M. pulcherrima as a Biocontrol Agent in Postharvest Fruit

According to estimates from the Food and Agriculture Organization (FAO), postharvest losses of fruits and vegetables account for 44% of production. These losses can occur throughout the production chain due to improper handling at harvest, inadequate temperatures during transport and storage, or pathogen attacks [91].

Currently, the primary and most traditional treatment for postharvest fruit diseases is the pre- and postharvest application of synthetic fungicides. Continued use of these products can lead to pathogen resistance, requiring increasingly higher concentrations for control, as well as risks of soil and groundwater contamination and the accumulation of residues in food [1]. Emerging technologies such as postharvest biocontrol offer an alternative to traditional methods like fungicides, as they are safe for both the population and the environment [1].

**Figure 6:**
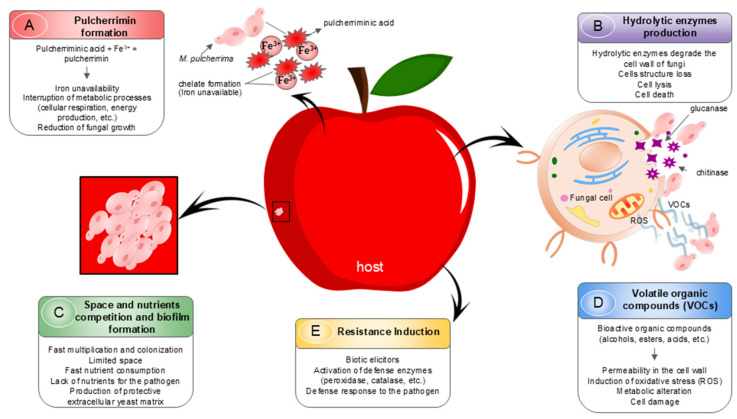
Biocontrol mechanisms of *M. pulcherrima* and its simultaneous effects on the fungal cell and the host (created using Inkscape v.1.4.3).

Studying and understanding the mechanisms of action of biocontrol agents is important for developing biopesticide formulations based on antagonistic yeasts, as it helps select the ideal strains for each specific type of fruit [9,41]. Over the past 10 years, different strains of *M. pulcherrima* have been studied as biocontrol agents in postharvest fruit, and their various modes of action have been described. *M. pulcherrima* yeasts, used as biocontrol agents, can inhibit pathogens through various antagonistic mechanisms, including competition for space and nutrients—mainly via pulcherrimin production—biofilm formation, the production of VOCs and hydrolytic enzymes, and the induction of resistance in fruits, as shown in Figure 6.

These yeasts have demonstrated antagonistic activity against several fungi that cause postharvest diseases, including the causal agents of gray mold on apples, grapes, blueberries, and strawberries (*B. cinerea*); green and blue mold on citrus and apples (*P. digitatum* and *P. expansum*); anthracnose on mango (*C. gloeosporioides*); and black spot on grapes, strawberries, and jujube fruits (*A. alternata*), among others, as shown in Table 3.

As shown in Table 3, research on the fungus *B. cinerea*, which causes gray mold in various fruits such as strawberries, grapes, and blueberries, using *M. pulcherrima* as a biocontrol agent, has generated significant interest among researchers. Mechanisms of action such as competition for nutrients and space, the induction of resistance by VOCs, and the activity of hydrolytic enzymes have been shown to increase the protection of fruits against gray mold development [20,32,66,67]. Other relevant fungi in studies using *M. pulcherrima* as a biocontrol agent include *P. expansum*, *P. italicum*, and *P. digitatum*, which are responsible for blue mold and green mold, respectively, in apples and citrus. The best biocontrol results for these fungi are attributed to mechanisms such as iron depletion (pulcherrimin) in grapes and apples [4,22,36], as well as the production of hydrolytic enzymes and biofilm formation in citrus [32,47].

Just as the interaction among the yeast, pathogen, and host is crucial for successful biocontrol [46], the interaction among environmental conditions (such as temperature and humidity), the biocontrol agent, and the host’s physiology can also determine optimal outcomes in biological control. Another important consideration in biological control is the method and timing of treatment application, as the application technique and the concentration of cells in the biopesticide can affect the colonization capacity, development, and survival of these biocontrol agents [5]. For example, selecting *M. pulcherrima* strains that can survive and develop in low-temperature environments, such as cold storage chambers commonly used in food storage to extend shelf life, illustrates how the interaction among environment, biocontrol agent, and host should function [37].

The effectiveness of *M. pulcherrima* biocontrol also depends on the availability of iron in the substrate for its growth or in the host it will colonize, since its main biocontrol mechanism is the ability to form pulcherrimin, which depends directly on this nutrient [33].

#### 3.3.1. Competition

One of the main mechanisms of action used by microorganisms that act as biocontrol agents is competition. This mechanism involves competition between the antagonist and the phytopathogen for resources essential to the survival of both, such as physical space and nutrients needed for their development [59,88].

Epiphytic yeasts make up the largest fraction of the microbiota on the surfaces of fruits and vegetables. Their frequent colonization, adaptation to diverse environmental conditions, and rapid growth make them ideal candidates for use as biocontrol agents [48,92].

Yeasts of the genus *Metschnikowia* are commonly found on the surfaces of fruits and leaves, in juices, and even in soil. Some strains of *Metschnikowia* sp. have been studied for their potential use in biocontrol. One of their most significant and well-known mechanisms of action is competition for nutrients, especially iron ions, which are essential for the development of microorganisms. This competition provides an advantage in suppressing various pathogens that cause plant diseases when used in biocontrol [60].

Yeasts of the *M. pulcherrima* clade can produce a secondary metabolite called pucherrimic acid (2,5-diisobutyl-3,6-dihydroxypyrazine-1,4-dioxide), which is formed by the oxidation of cyclodileucine (Leu-Leu cycle) [78,93]. The capacity and level of production of this acid vary among yeast species and strains [33,78]. According to Gore-Lloyd et al. [33], the SNF2 transcriptional gene in *M. pulcherrima* regulates the antifungal activity and biosynthesis of pulcherriminic acid.

**Figure 7:**
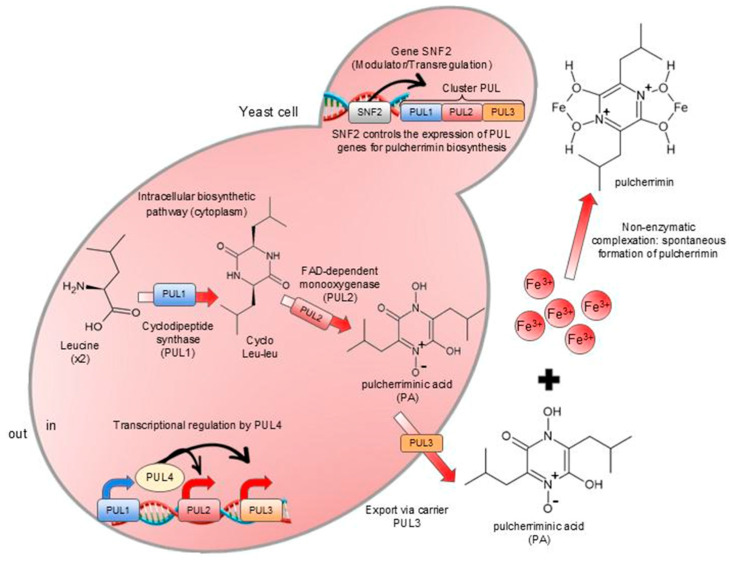
Biosynthesis, secretion, and formation of pulcherrimin (created using ACD/ChemSketch, v. 2025. 2.0).

Pulcherriminic acid is a water-soluble molecule produced inside the yeast cell, and, when secreted, it is able to chelate Fe^3+^ ions present in the extracellular medium, forming reddish, insoluble chelate complexes that precipitate. The immobilization of these iron ions reduces their bioavailability for assimilation by other microorganisms. This mechanism is often associated with the antagonist activity of this yeast, but its effect depends on the yeast species and strain studied [14,33,62,93], as shown in Figure 7, adapted from Krause et al. [94], which illustrates the formation of these complexes, called pulcherrimin. Its production is also associated with a change in the color of yeast colonies, giving them a reddish hue that intensifies with higher concentrations of iron ions. When secreted, the pigment forms a visible halo of the same color around the colonies [30].

Although the antagonistic potential of *M. pulcherrima* is often been attributed to the production of pulcherriminic acid and the resulting competition for iron, some studies show that strains not producing this compound can also antagonize certain species of filamentous fungi [33].

Gore-Lloyd et al. [33] studied spontaneous mutant strains of *M. pulcherrima* APC 1.2 (W8, W10, and W11) that were unable to produce pulcherrimin due to a point mutation in the gene encoding METSCH-Snf2. They found that even without this compound, the strains exhibited antagonistic behavior. Strain W8 inhibited the growth area of the fungus *Botrytis caroliniana* by 80% in a binary competition assay on PDA culture medium plates. In competition tests against *Gibberella fujikuroi* and *F. oxysporum*, W8 inhibited 56% and 45% of their growth areas, respectively, while W10 and W11 reduced the growth areas of the same fungi by 37% to 51%. However, when W8 was applied to cherries to evaluate control of *B. caroliniana* symptoms, its performance was poor, as it was unable to prevent mycelial development of the fungus, resulting in a considerable increase in the diameter of the rot.

Five strains of *M. pulcherrima* that showed strong inhibitory potential against the development of fungal colonies were tested by Steglinska et al. [30] to assess their efficiency in pulcherrimin formation. The yeast growth medium (YPD) was supplemented with three concentrations of FeCl_3_ (5, 10, and 20 μg·mL^−1^). The authors observed that the halos formed by pulcherrimin production decreased in diameter as the FeCl_3_ concentration in the culture medium increased; however, the intensity of the halo coloration increased proportionally, due to the higher production of this compound.

Yang et al. [46] tested the effect of adding 0, 5, 10, 15, and 20 μg·mL^−1^ of FeCl_3_ to PDA on the production of pulcherrimin by *M. pulcherrima* E1 and its inhibitory effect on the growth of *Pestalotiopsis vismiae*, a fungus that causes rot symptoms in loquats. The authors observed that both the inhibition diameter and the diameter of the halo formed around the colony decreased as the iron concentration in the medium increased, becoming nonexistent with the addition of 20 μg·mL^−1^. The inhibition zones of *P. vismiae* without supplementation and with the addition of 5 μg·mL^−1^ of iron were 5.9 ± 1.19 mm and 4.3 ± 0.65 mm, respectively. In the medium with 20 μg·mL^−1^ of FeCl_3_, the inhibition zone was 0.8 ± 0.42 mm.

Competition is one of the biological control mechanisms of these yeasts, serving as a passive and effective strategy that can sometimes resemble the disease control effect of a synthetic fungicide [60]. Competition for nutrients such as iron is not the only mechanism used by *M. pulcherrima* yeasts; they also compete for space. This competition is closely related to the initial concentration of yeast cells inoculated in the inhibition tests [5].

*M. pulcherrima* 34-UEM at concentrations of 10^5^ and 10^6^ cells·mL^−1^ completely inhibited the germination of *B. cinerea* spores (10^6^ CFU·mL^−1^). However, at concentrations lower than 10^5^ cells·mL^−1^, this inhibition decreased to 66%, indicating that the effect depends on yeast cell concentration [5].

Another important aspect of the competition mechanism, according to Oro et al. [52], is the ability to colonize the fruit surface within the first 24 h after application, which can determine the effectiveness of biocontrol agents. The viability of *M. pulcherrima* on the surface of certain fruits after artificial inoculation has been tested a few times and has shown good results. *M. pulcherrima* exhibited exponential growth over 96 h on the surface of mandarin fruits [10], increasing its population density by 22% during the colonization of injured fruits. In a similar experiment, artificially wounded mandarin and grape berries were inoculated with *M. pulcherrima* cells (10^8^ cells·mL^−1^) and reached high levels of colonization by the yeast after 96 h, demonstrating its strong colonization and adaptation capacity [44].

#### 3.3.2. Production of Volatile Organic Compounds

Some microorganisms can produce volatile organic compounds (VOCs) with antimicrobial effects. VOCs are low-molecular-weight compounds generated by the secondary metabolism of certain bacteria, fungi, and yeasts. These molecules can diffuse through the air and cross physical barriers such as membranes due to their high vapor pressure at room temperature, and they can be used as biocontrol agents in agriculture, industry, and pharmacology [16,66].

Alcohols, ketones, esters, hydrocarbons, terpenes, and sulfur compounds are among the chemical groups of VOCs identified from these microorganisms. Some of these compounds have demonstrated antimicrobial, antifungal, insecticidal, and phytomodulatory activity [16], as shown in Table 4. In postharvest biological control, VOCs offer a significant advantage because their action does not require direct contact with the pathogen or the fruit surface, making their application method especially beneficial [95].

Tests on the action of VOCs produced by *M. pulcherrima* have been conducted on various fruits, including strawberries [16,44,45], mandarins [10,44,45], grapes [5,14,44], blueberries [66,67], and apples [21].

Generally, these tests use culture medium plates without lids containing grown *M. pulcherrima* cultures, which are placed inside containers with the fruits. The containers are then sealed, forming a chamber that retains the VOCs produced by the yeasts during their development. The effect of these compounds is evaluated after a few days by analyzing the number of fruits that developed disease symptoms in these containers and the severity of the disease [5,14,16,44].

Sabaghian et al. [29] quantified the profiles of volatile compounds produced by *M. pulcherrima* and observed that alcohols such as isobutyl alcohol, isoamyl alcohol, phenylethyl alcohol, and isoprenyl alcohol were present at higher levels. They also verified the presence of low-molecular-weight organic acids (acetic acid, isovaleric acid, n-caprylic acid, pelargonic acid, and n-capric acid) and volatile ethyl esters (lauric acid ethyl ester). Oztekin and Karbancioglu-Guler [5], in an in vitro study on the inhibition of mycelial growth of *B. cinerea* by VOCs produced by *M. pulcherrima* 34-UEM, observed a 42.66% inhibition of fungal growth when the yeast was cultivated in YEPD solid medium at pH 4.5, and 18.99% inhibition at pH 6.0. In a similar experiment, the *M. pulcherrima* E1 strain grown in NYDB liquid medium (pH 4.5) also inhibited the mycelial growth of *B. cinerea* by 47.48% through the production of VOCs [14].

Oro et al. [16] analyzed the production of VOCs from liquid cultures of 6-day-old *M. pulcherrima* Disva 267 using microextraction and gas chromatography. They found that the most abundant compound detected on the sixth day was ethyl acetate (115 mg·L^−1^). In addition, isoamyl alcohol, amyl alcohol, 2-phenyl-ethanol, isobutanol, isoamyl acetate, ethyl hexanoate, acetaldehyde, and ethyl butyrate were also detected in small quantities. In vitro, these compounds significantly reduced the mycelial growth of *B. cinerea* (56%) and *Monilinia fructicola* (42%), while reductions in less than 10% were observed in *A. alternata*, *Aspergillus carbonarius*, and *Cladosporium* spp.

In Yang et al. [46], thirteen volatile compounds were produced by the E1 strain of *M. pulcherrima*. Among these, 3-methyl-1-butanol was the most abundant, accounting for 56.47% of the total peak area in the chromatogram of the VOCs sample. Ethyl acetate, phenylethyl alcohol, 2(3H)-Furanone, dihydro-5-pentyl and 1-hexanol were present in moderate concentrations. Other compounds, such as hexanoic acid ethyl ester, 1-pentanol, 2-nonanone, 1-heptanol, and 1-octanol, were found at lower concentrations, each representing less than 1.0% of the total peak area in the chromatogram. Based on these results, the authors selected some compounds with the highest concentrations (3-methyl-1-butanol, phenylethyl alcohol, 1-hexanol, and ethyl acetate) and separately evaluated, in vitro, the concentrations of standards of these compounds capable of inhibiting the growth (IC50) of *P. vismae* by 50%. They observed that, to achieve the IC50 for this fungus, which causes rot in loquat fruits, concentrations of 34.19 μL·L^−1^ of 3-methyl-1-butanol, 20.41 μL·L^−1^ of phenylethyl alcohol, 11.76 μL·L^−1^ of 1-hexanol, and 280 μL·L^−1^ of ethyl acetate were required.

A synergistic effect of VOCs produced by *M. pulcherrima* E1 inhibited the growth of *P. vismae* by up to 41.79% after two days of cultivation on double plates compared to the control, indicating a fungistatic rather than fungicidal potential for these compounds, according to Yang et al. [46]. In Marsico et al. [31], VOCs produced by *M. pulcherrima* Ale4 and N20/006 (10^7^ cells·mL^−1^) reduced the mycelial growth of *B. cinerea* in vitro by 60.4% and 61.2%, respectively, compared to the control treatment.

Regarding in vivo results, the VOCs produced by *M. pulcherrima* T-2, as studied by Li et al. [67], increased the concentration of flavonoids in blueberry fruits. Flavonoids are essential as an initial barrier against pathogen action in vegetables. The VOCs also improved the control of gray mold in these fruits. This effect is possibly linked to the induction of resistance in the fruits caused by exposure to these compounds [67].

Parafati et al. [45] immobilized *M. pulcherrima* MPR3 yeast cells in hydrogel spheres and studied the effect of VOCs produced by these yeasts both in vitro and in vivo. In the in vitro tests, the authors evaluated the efficiency of VOCs on the mycelial growth of the fungi *B. cinerea*, *P. digitatum*, and *P. italicum* by placing about 50 hydrogel spheres inoculated with yeast in Petri dishes and covering them with another Petri dish containing PDA medium inoculated with 20 µL of spore solution with 10^6^ CFU·mL^−1^. Both dishes were sealed with parafilm and stored at 20–25 °C. This resulted in a reduction of 65–67% in mycelial growth for all the fungi tested, compared to the control treatment (spheres without yeast). In the in vivo test, some fruits were artificially inoculated: strawberries (with *B. cinerea*) and tangerines (with *P. digitatum* and *P. italicum*, separately). The fruits were placed in sealed polypropylene packages and stored at 20–25 °C, with hydrogel spheres containing the yeasts at the bottom of each package. The action of VOCs produced by *M. pulcherrima* MPR3 was not as effective in controlling the incidence of gray mold on strawberries and blue and green molds on tangerines. However, compared to the control, treatments with VOCs significantly reduced disease severity and lesion diameters for gray and green mold diseases.

#### 3.3.3. Action of Hydrolytic Enzymes

Hydrolytic enzymes produced by microorganisms, such as certain yeasts, can break down larger molecules into smaller ones through hydrolysis. The production of these enzymes by *M. pulcherrima* is another biocontrol mode of action for these yeasts, as they degrade components of fungal cell walls—mainly chitin, mannoproteins, cellulose, β-1,3- and β-1,6-glucans—thereby limiting fungal development or even causing cell death [5,30].

*M. pulcherrima* has a history of producing various enzymes, including chitinase, cellulase, pectinase, gelatinase, and β-1,3-glucanase [32,62]. These enzymes may be produced individually or simultaneously, depending on the strain, and can act together to structurally damage the pathogen’s cells [32]. According to Oztekin and Karbancioglu-Guler [32], pectinase-producing yeasts should be used carefully when applied to fruit surfaces, as they may degrade the plant cell wall, potentially sensitizing the fruit tissue and making it more susceptible to colonization by pathogens.

Chitinase, cellulase, and gelatinase were the enzymatic activities present in all *M. pulcherrima* yeasts tested (4-UDM, 21-KTM, 26-BMD, 32-AMM, and 34-UEM) by Oztekin and Karbancioglu-Guler [32] in in vitro tests. Protease and pectinase were also present in 21-KTM and 32-AMM, and β-1,3-glucanase was found in these two strains as well as in 34-UEM. Steglinska et al. [30] detected α- and β-glucosidase in *M. pulcherrima* strains, which act on fungal membranes and cause cell death. Additionally, the production of acid phosphatase, cystine, valine, and leucine arylamidase, esterase lipase (C8), and esterase (C4) was observed in the same yeasts, with leucine arylamidase being directly involved in pulcherrimin production [93].

In Marsico et al. [31], *M. pulcherrima* strains N20/006, C, and Pr7 produced various extracellular enzymes in vitro at both 25 °C and 0 °C. All three strains produced lipase; N20/006 also produced pectinase and protease, while Ale4 produced β-1,3-glucanase and pectinase. Oztekin and Karbancioglu-Guler [5] examined the enzymatic profile of *M. pulcherrima* 34-UEM, which showed activity for β-1,3-glucanase, cellulase, protease, and gelatinase; β-glucosidase and leucine arylamidase were produced in higher amounts (≥40 nmol).

#### 3.3.4. Mycocins (Killer Toxins)

Mycocins, also known as killer toxins, are secondary metabolites such as proteins or glycoproteins produced by fungi and yeasts. They can interfere with the development and survival of sensitive strains of phylogenetically related species [96]. As with other biocontrol strategies mentioned above, the production mycocins by *M. pulcherrima* yeast has been reported as one of the biocontrol modes of action of these yeasts [32].

In Tian et al. [24], the authors observed that *M. pulcherrima* produced non-volatile antimicrobial substances. After yeast cells were grown in liquid PD medium, the culture was sterilized at 121 °C for 20 min, and a filtrate obtained from this medium was applied to mango fruits artificially inoculated with *C. gloeosporioides*. There were no significant differences between these two treatments and the control; both were effective in controlling anthracnose symptoms. When the treated fruits were stored at 15 °C, the inhibition rate was 24% for fruits treated with the yeast suspension and 23% for those treated with the filtrate. For fruits stored at 25 °C, the inhibition rates were 31% and 39%, respectively [24]. In contrast, cell-free filtrates of *M. pulcherrima* in Steglińska et al. [30] did not show any antimicrobial activity, demonstrating that different strains can influence the production of secondary metabolites such as killer toxins.

Yeasts of the species *M. pulcherrima* (PC-1-48.2 and PA-6-26.1), *M. fructicola* (PC-6-12.1, PA-6-34.1N, and PC-6-17.1), and *M. sinensis* (PC-6-49.1) were isolated from freshly harvested sweet and sour cherries in Stanevičienė et al. [48]. These strains were tested for killer activity using an in vitro assay with a sensitive yeast strain, *Saccharomyces cerevisiae* (BY4741). The sensitive yeast was grown on a plate with Methylene Blue Azide (MBA) culture medium, and a drop of the culture medium from each *Metschnikowia* yeast with killer potential was inoculated onto the growth. The appearance of clear inhibition zones, indicating the lysis of *S. cerevisiae* cells by killer toxins. *M. pulcherrima* PA-6-26.1 showed moderate killer activity against sensitive yeasts, while *M. pulcherrima* PC-1-48.2, *M. fructicola* PC-6-17.1, PA-6-34.1N, PC-6-12.1, and *M. sinensis* PC-6-49.1 exhibited weaker killer activity against the same yeast. In addition to tests with *S. cerevisiae*, other target yeasts were tested for sensitivity to killer activity, including *Rhodotorula graminis*, *R. glutinis*, *Candida albicans*, *C. guilliermondii*, *Sporobolomyces roseus*, *Cryptococcus wieringae*, and *Aureobasidium pullulans*. The results showed that strains of *M. pulcherrima*, *M. fructicola*, and *M. sinensis* exhibited broad antagonistic activity against the growth of all tested yeasts, with the smallest inhibition zone being 1 mm for *C. wieringae* and *S. roseus*, and the largest inhibition zones (5–3 mm) observed in *A. pullulans*, *R. graminis*, and *R. glutinis*, produced by *M. pulcherrima* PA-6-26.1 and *M. fructicola* PC-6-12.1 and PA-6-34.1N.

Marsico et al. [31] tested three isolates of *M. pulcherrima* (Ale4, N20/006, and Pr7) using a technique in which a layer of cellophane was placed between the agar and the yeast cells inoculated on the cellophane. After inoculation, the plates were incubated for 2 days at 25 °C. After this period, the cellophane disks were removed, and agar disks with *B. cinerea* growth were placed in the center of the plates, which were then incubated again to evaluate the influence of secondary metabolites produced by these yeasts. These metabolites crossed the cellophane pores, were deposited and diffused in the agar, and inhibited the mycelial growth of the fungus. The authors state that all three strains produced fungistatic secondary metabolites, as evidenced by their ability to completely inhibit the mycelial growth of *B. cinerea*.

Among the biocontrol mechanisms, the production of killer toxins has the least factual evidence described in the articles selected for this review, because these compounds produced by *M. pulcherrima* are associated with other antagonistic factors, such as the production of pulcherrimin [24]. The lack of purification and biochemical characterization of these compounds hinders a full understanding of their functions.

#### 3.3.5. Biofilm Formation

Some microorganisms, such as bacteria and yeast, can produce a polymeric extracellular matrix composed of carbohydrates, proteins, lipids, nucleic acids, and water on both biotic and abiotic surfaces, where clusters of cells remain, called biofilm [32,97]. The production of this biofilm is originally a strategy that enables these microorganisms to survive in hostile environments and increases their resistance [98]. However, the ability to produce these exudates can also serve as a biocontrol mechanism against phytopathogenic species, contributing to the efficient colonization of these antagonists on plant surfaces and hindering the development of disease-causing microorganisms [14,32].

The biofilm formation capacity of the yeast strain *M. pulcherrima* E1 was evaluated using the optical density (OD) assay at 590 nm as described by Yang et al. [46], where a higher OD value indicates greater biofilm production. NYDB medium was used to grow yeast cells in 96-well polystyrene plates in vitro, and biofilm formation was measured over 72 h at 3, 24, 48, and 72 -hour intervals. Biofilm formation was also assessed in the pulp of some loquat fruits artificially inoculated with *M. pulcherrima* and incubated for 24 h, using scanning electron microscopy (SEM). The OD results showed that *M. pulcherrima* cells strongly adhered to the 96-well plates from 3 h of incubation (OD 1.12), but over time, cell adhesion and biofilm formation gradually decreased until 72 h of incubation (OD 0.73 ± 0.03). Similarly, the micrographs demonstrated the strong potential of these yeasts to form biofilms on the surface of loquat fruit tissues.

In Parafati et al. [14], *M. pulcherrima* MPR3 exhibited higher levels of biofilm production after 72 h (OD 1.12 ± 0.16). Three different strains of *M. pulcherrima* (DN-HS, DN-MP, and DN-UY) also showed varying levels of biofilm formation after 72 h (1.27 ± 0.33; 0.73 ± 0.04; and 0.32 ± 0.09, respectively) in Acar et al. [22]. In Oztekin et al. [5,32,47], *M. pulcherrima* 26-BMD, *M. pulcherrima* 34-UEM, and *M. aff. pulcherrima* P01A016 32-AMM exhibited lower levels of biofilm production after 72 h (OD 0.69 ± 0.14; 0.43 ± 0.03; and 0.66 ± 0.08, respectively). A positive relationship between biofilm formation and the colonization of grape berry wounds by *M. pulcherrima* MPR3 was also observed in the in vivo tests of Parafati et al. [14].

#### 3.3.6. Resistance Induction

The use of yeasts as biological control agents extends beyond their direct action on plants or fruits. Biotic elicitors are molecules or biological agents that can trigger defense responses in plants, acting as activators of the plant immune system [99]. Applying yeast as a biocontrol agent can function as an elicitor, stimulating responses to stresses caused by pests and diseases, such as increasing the activity of enzymes involved in resistance induction [21,24,44].

According to Parafati et al. [44], the interaction among yeast, pathogen, and fruit—also called tritrophic interaction—is directly related to biocontrol activity. Therefore, the activation of enzymes in fruits such as peroxidase (POD) and superoxide dismutase (SOD), which are involved in the plant defense system, may be one of the modes of action of biocontrol. Other enzymes associated with fruit defense against pathogens, such as polyphenol oxidase (PPO), catalase (CAT), ascorbate peroxidase (APX), and phenylalanine ammonia-lyase (PAL), have also been documented, showing activity in response to stimuli generated after treatment with biocontrol agents [46].

*M. pulcherrima* Mp-30 yeast cells were applied to apple fruits using two methods to evaluate their resistance-inducing effect: immersing the fruits in a cell suspension (10^8^ cells·mL^−1^) for 5 min and inoculating artificially injured fruits with 20 μL of cell suspension (10^8^ cells·mL^−1^). After 72 h, the same fruits were inoculated with *B. cinerea* next to the yeast wounds [21]. According to the results, there was no difference between the lesion sizes on treated and control fruits, indicating no induction of resistance in the fruits. In contrast, Yang et al. [46] found that POD and PPO enzyme activities increased over the storage period (8 days) in injured and artificially inoculated loquat fruits, regardless of treatment. In fruits treated with *M. pulcherrima* E1, these enzymes showed higher activity than in control fruits. CAT and APX enzyme activities were higher in treated fruits only during the first few days and gradually decreased throughout storage. In control fruits, a rapid decrease in these enzyme activities was observed from the third day of storage. PAL activity was significantly higher and remained constant over time in treated fruits compared to control fruits, with a slight initial increase followed by a decrease.

The difference between the results found in these two studies may be related to the specific characteristics of each *M. pulcherrima* strain (Mp-30 and E1). The Mp-30 yeast may not act as an elicitor, while the E1 strain may have this potential. Additionally, the fruits and pathogens used in each experiment have distinct biological characteristics and, consequently, specific responses to external stimuli. This demonstrates the interdependence of the interaction among yeast, pathogen, and host [44].

Parafati et al. [44] evaluated the activity of POD and SOD enzymes in the peel of tangerines treated with *M. pulcherrima* MPR3. The authors observed that POD activity was strongly induced by the application of yeast, and, compared to the control, the activity of this enzyme was significantly higher, reaching its maximum in treated fruits after 24 h. The SOD enzyme showed less significant activity than POD but was still significantly higher in treated fruits than in control fruits after 24 h, when the peak activity of this enzyme occurred. After this time, SOD activity decreased and became almost constant, while activity in control fruits gradually increased, reaching its maximum at 72 h. This demonstrates the possibility of interrupting disease development earlier in treated fruits than in control fruits due to the induction of resistance in the fruits.

### 3.4. Synergistic Effect of Combining Various Emerging Ecological Techniques with Biocontrol Using M. pulcherrima

Previous sections of this review demonstrated the effectiveness of using *M. pulcherrima* alone for the biological control of fungi and postharvest disease symptoms in various foods affected by pathogen contamination. However, some authors agree that relying on a single method for the prevention and control of postharvest diseases is insufficient to completely prevent their occurrence, as the duration of effectiveness is often limited [4].

Some researchers have investigated the combined use of emerging, ecological, and safe techniques to control these diseases, along with the biological control provided by *M. pulcherrima*, and their results have been promising. Several studies report a synergistic effect from combining biological control with other methods, which enhances the inhibition of these microorganisms and diseases, reduces the rate of infection and reinfection caused by several important phytopathogenic fungi, and increases the shelf life of these foods [4,13,37,47,58,59].

Edible coatings or coverings are thin layers of biopolymeric, biodegradable, and biocompatible material—made from proteins, polysaccharides, and/or lipids—applied directly to food, commonly by immersion or brushing [100,101]. The coating formulation must consider the characteristics of the food to be coated and the intended function of the coating. These coverings serve as physical protective barriers, limiting gas exchange (O_2_, CO_2_, and ethylene), reducing mass and firmness loss, and consequently increasing shelf life [102]. Additionally, various antimicrobial substances, such as essential oils, nanoparticles, plant extracts, and antagonistic microorganisms, can be incorporated into the polymeric matrix to help prevent postharvest diseases [103].

Parafati and colleagues [44] conducted a study to determine the effect of an edible coating based on locust bean gum (LBG) on the survival and biocontrol capacity of yeasts. The authors added the yeasts *Wickerhamomyces anomalus*, *M. pulcherrima*, and *Aureobasidium pullulans* at a concentration of 10^9^ cells·mL^−1^, polymeric solutions of LBG (0.5% and 1%), and sterile distilled water (control), and studied the viability and bioactivity of these coatings applied to tangerine fruits by immersion. Each fruit was artificially inoculated with 20 µL of a spore suspension of the fungi *P. digitatum* and *P. italicum* (10^5^ CFU·mL^−1^) and stored for 5 days (25 °C; 95% RH). The authors observed that both the use of yeast alone and yeast incorporated into the coatings controlled the development of pathogens in the fruits. When comparing the two, coatings with LBG were more effective, as they increased the survival of yeasts on the tangerine peel, prolonging their viability. The coating (LBG 1%) containing *M. pulcherrima* showed the best results in controlling deterioration by green mold. For the control of blue mold, coatings with this same yeast were equally effective, regardless of LBG concentration. The authors indicated that stimulation of peroxidase (POD) and superoxide dismutase (SOD) activity in the tangerine peel is the possible biocontrol mechanism of this yeast.

Settier-Ramírez et al. [35] applied edible coatings made from a matrix of apple pomace residues (pulp, peel, seeds, and lignified parts) and incorporated *M. pulcherrima* yeast, isolated from apple peels, into these coatings. The authors applied these coatings to apple fruits to limit the development of blue mold (*P. expansum*), which was artificially inoculated on the fruit surfaces. The coating delayed disease development in fruits stored at 21 °C for 17 days, due to yeast colonization at the inoculation site. Using the coating with yeasts supported their survival and viability, thanks to its good adherence to the fruit and the matrix composition (water-soluble and water-insoluble fractions with sugars, polysaccharides, and fibers), which likely provides nutrients for yeast growth and increases resistance to dehydration in these microorganisms.

The use of a controlled and modified atmosphere in packaging is based on altering the concentrations of CO_2_ and O_2_ inside the package. When the modified atmosphere packaging (MAP) technique is used, the air composition inside the packaging changes due to the balance between food respiration, which consumes much of the available O_2_ and releases CO_2_, and the permeability of the packaging [104]. This method reduces the deterioration of fresh food, as the high concentration of CO_2_ inhibits the growth of microorganisms, providing an alternative to synthetic pesticides [104]. Saravakumar et al. [105] demonstrated the effectiveness of combining controlled atmosphere and bioprotection with *M. pulcherrima* MACH1. Fruits artificially inoculated with *B. cinerea*, *A. alternata*, and *P. expansum* (10^5^ CFU·mL^−1^) and treated with *M. pulcherrima* (immersion in a solution with 10^7^ cells. mL^−1^ for 60 s) were stored for 8 months in a cold chamber (1 °C) with a controlled atmosphere of either 2% O_2_/3% CO_2_ or 1% O_2_/2% CO_2_. In a chamber with 2% O_2_/3% CO_2_, the treatment with *M. pulcherrima* resulted in 25.2% of the fruits showing symptoms of rot, which was 61% fewer than in the control treatment. In the environment with 1% O_2_/2% CO_2_, the treatment with *M. pulcherrima* did not differ statistically from the control.

De Paiva et al. [40] studied the use of MAP in combination with antagonistic yeasts. Cleaned sweet cherries were wounded and inoculated with a suspension of yeast cells (*M. pulcherrima* L672 or *H. opuntiae* L479, approximately 10^8^ cells·mL^−1^), followed by a suspension of phytopathogen spores (*P. expansum* M639). Controls were inoculated with either distilled water and phytopathogen or distilled water alone. The inoculated cherries were then placed in polyethylene trays and sealed with three different 40 μm thick polypropylene films: macroperforated film with six holes (9 mm in diameter); BOPP microperforated film with 16 holes (100 μm in diameter); and BOPP microperforated film with three holes (100 μm in diameter). The trays were stored at 1 °C and 90–95% relative humidity in the dark and evaluated for 35 days. The highest average CO_2_ concentration in the headspace was observed after 35 days of storage in packaging with BOPP film and three holes (11.25 kPa). According to the authors, previous studies indicate that at a CO_2_ concentration of 5 kPa, this gas exhibits fungistatic action under low O_2_ tension. The 5 kPa concentration was reached in these packages between 7 and 21 days of storage. Although both yeasts survived and grew in MAP packaging stored at low temperature, a higher concentration of *M. pulcherrima* was detected in the packaging where it was applied, demonstrating its superior adaptation to modified atmosphere packaging. The most effective treatment, which resulted in the lowest growth of the pathogen inoculated in sweet cherries, was packaging with BOPP microperforated film with three holes and inoculation with *P. expansum* and *M. pulcherrima*, which delayed the appearance of mold for up to 28 days of storage.

To evaluate the effect on the shelf life of sweet cherries, Cabañas et al. [55] used a combined application of MAP packaging and antagonistic yeasts (*M. pulcherrima* and *Pichia kudriavzevii*). Treatments on cherry fruits included immersion for 5 min in water at 5 °C (negative control), individual yeast solutions dispersed in water at 5 °C (10^8^ cells·mL^−1^), and a solution of the fungicide fludioxonil (2.5 mL·L^−1^) at 5 °C (the positive control). After drying, the fruits were placed in passive modified atmosphere bags (~1.5 kPa O_2_; ~9 kPa CO_2_), sealed with elastic, and stored in a cold room (2 °C and 90–95% RH) for 40 days. The combination of MAP and yeast application effectively controlled the incidence of fruit rot during cold storage, with average values of 35.37 ± 3.57% for *M. pulcherrima* and 31.31 ± 6.98% for *P. kudriavzevii*, while the negative control and fungicide treatments showed 53.60 ± 16.32% and 23.36 ± 3.96% incidence of rot, respectively, at the end of 40 days. The use of MAP resulted in less mass loss over time, and a slight decrease in volatile compounds associated with the aroma of fresh fruits was observed compared to the control treatment.

Elicitors are natural or synthetic substances that, when applied to plants or fruits—even after harvest—trigger a series of direct or indirect natural defense responses, such as enzyme activation, cell wall strengthening, and increased resistance to pathogens [99].

Shao et al. [25] studied the synergistic effect of applying *M. pulcherrima* combined with elicitors to control anthracnose symptoms and maintain the quality of mango fruits during postharvest storage. A mixture of *M. pulcherrima* suspension (10^8^ cells·mL^−1^), salicylic acid (SA) solution at 50 mg·L^−1^, and calcium chloride (CaCl_2_) solution at 1.0 g·L^−1^ was applied to mango fruits by immersion. Each component of the mixture and distilled water (control) was also applied separately to other fruits. After treatment, the fruits were stored at 25 °C and 90–95% relative humidity. Once dried, analyses were conducted every 4 days to determine the disease index, perform physicochemical analyses, and measure the activities of the enzymes polyphenol oxidase (PPO), phenylalanine ammonia lyase (PAL), chitinase (CHT), and β-1,3-glucanase (GUN) in mango samples. The combination of yeast, SA, and CaCl_2_ was more effective than the individual treatments, preventing anthracnose development and delaying fruit maturation after 24 days of storage, as indicated by a lower a* color coordinate, higher average firmness, and lower total soluble solids content. Additionally, fruits from this treatment had a higher vitamin C concentration and increased activities of defensive enzymes such as PAL, PPO, CHT, and GUN.

Calcium propionate (CaP) is recognized by the Food and Drug Administration (FDA) and the European Food Safety Authority (EFSA) as a safe food additive for consumption, and serves as an antimicrobial preservative for foods [106,107].

To evaluate the effect of using CaP (2% *w*/*v*) in conjunction with the yeast *M. pulcherrima* in controlling the development of rot symptoms in jujube fruits, Guo et al. [59] prepared suspensions of *M. pulcherrima* with 2% *w*/*v* CaP at three different cell concentrations (10^8^, 10^7^, 10^6^, or 0 cells·mL^−1^). Artificially wounded jujube fruits were inoculated with these suspensions, and after 2 h, 20 μL of a suspension of *P. citrinum* or *A. alternata* spores (10^4^ CFU·mL^−1^) was dispensed into the same wounds. The dried fruits were stored at 24 °C and 95% RH. According to the authors, the growth of *M. pulcherrima* was not affected by the addition of CaP, and increasing the concentration of yeast cells in the treatment resulted in greater control of the incidence of blue mold and alternaria rot, as well as a reduction in lesion size. Compared to control treatments (with *M. pulcherrima* and without CaP), combined treatments (with *M. pulcherrima* and 2% *w*/*v* CaP) reduced disease incidence by 16.8–27.3% for *P. citrinum* and by 25.2–31.4% for *A. alternata*. The authors also noted that the positive effects of the combined treatments were due to CaP directly inhibiting the development of *P. citrinum* and *A. alternata*, and possibly because *M. pulcherrima*’s mode of action was competition for nutrients and space.

Pedrozo et al. [4] used another food additive together with *M. pulcherrima* yeast to control blue mold symptoms in grapes. The study involved spraying a solution of sodium bicarbonate (NaHCO_3_) at a concentration of 0.3% *w*/*v* in combination with a mixture of two strains of *M. pulcherrima* yeast (Mp22, Mp36~10^8^ cells.mL^−1^) on grape bunches, which were subsequently inoculated with a solution of *P. expansum* PSS6 spores (10^4^ CFU·mL^−1^). The combined use of NaHCO_3_ and *M. pulcherrima* strains reduced the severity of blue mold by 90% on table grapes stored for 30 days at 2 °C.

Exposing food to ionizing radiation is an emerging technique used to ensure food safety because it controls the growth of microorganisms that can cause food poisoning [108]. According to Jeong and Jeong [109], X-rays, electron beams, and gamma rays are examples of ionizing radiation, and their direct effect on pathogenic microorganisms is the degradation of genetic material, including DNA or RNA.

Guo et al. [13] tested the combined use of ultraviolet-C (UV-C) and *M. pulcherrima* (10^8^ cells·mL^−1^) as a postharvest treatment on winter jujube fruits. They observed that this treatment significantly reduced contamination (16%) and lesion size in fruits artificially inoculated with *A. alternata* (10^5^ CFU·mL^−1^) and stored at 22 °C for 7 days. Non-inoculated fruits that received the same treatment and were stored in a cold room (0 ± 1 °C) for 45 days, followed by 22 °C for 7 days, showed a low percentage of rot symptoms during storage at low temperatures (13%) and at higher temperatures (23%), and their quality parameters were not affected by the treatment.

Guo et al. [58] used a combination of microwaves and a suspension of *M. pulcherrima* to verify its antagonistic effect on natural jujube fruits artificially inoculated with *P. citrinum*. Initially, the jujube fruits, which were already injured and inoculated with *P. citrinum* (10^5^ CFU·mL^−1^), were placed in a microwave oven for 2 min and then left at room temperature to re-equilibrate. Aliquots of the *M. pulcherrima* suspension (10^8^ cells·mL^−1^) were pipetted into the wounds, and the fruits were subsequently stored at 25 °C for 5 days. Non-inoculated fruits were microwaved in the same way, then immersed in a solution of *M. pulcherrima* cells (10^8^ cells·mL^−1^) for 1 min at room temperature, and allowed to air dry. After drying, the fruits were stored at 2 ± 1 °C for 45 days, and then for 7 days at 22 °C to determine disease development under shelf conditions. Individual treatments and controls (treated with water) were also tested. In the inoculated fruits, the combination of treatments reduced the incidence of rotten fruits to 21%, with lesion diameters of 1.0 cm. In the individual treatments, the minimum incidence of rotten fruits was 36%, and the average lesion diameter was 1.38 cm (for the treatment using only *M. pulcherrima*). In reducing the natural development of spoilage, the percentage of spoiled fruits treated with the combination of microwave and *M. pulcherrima* was only 6.2%, compared to 28.6% in fruits treated with water alone, 11.6% in those treated with microwaves, and 12.3% in those treated with *M. pulcherrima*. Jujube fruits treated with microwaves, *M.* *pulcherrima*, or a combination of both showed a higher ascorbic acid content than the control.

### 3.5. Use of M. pulcherrima in Commercial Product Formulations

Traditional methods for controlling postharvest fungal diseases have included pre- and postharvest treatments with conventional fungicides such as imazalil, thiabendazole, fludioxonil, prochloraz, and their mixtures; the processing and disposal of infected and rotten fruits; sanitizing fruits with sodium hypochlorite; applying natural and synthetic waxes with added fungicides; and storing fruits at low temperatures [110].

The limiting factors for using commercial chemical fungicides are the health problems caused by these products and the residues they leave in fruits, soil, and groundwater, as well as the development of resistant microorganisms, which require even higher concentrations of fungicides for control. Furthermore, countries that import these fruits or products that are derived from their processing are increasingly concerned about consuming healthy products and are beginning to monitor maximum residue limits as a requirement for acceptability [110].

According to the United States Environmental Protection Agency (EPA), biopesticides are pesticides made from natural materials such as animals, plants, bacteria, and certain minerals [111]. The Food and Agriculture Organization of the United Nations (FAO) defines biopesticides as a generic term for substances of natural origin, including microorganisms, plants, and semiochemicals. These materials can be formulated and applied like conventional chemical pesticides and are typically used for short-term traditional control [110]. The regulation and marketing of these products depend on several criteria to ensure their safety and effectiveness [111].

To register a biopesticide in the European Union or the United States, certain criteria, which vary between the responsible authorities in these regions, must be met. In general, the main criteria for approval of active substances are the taxonomic identification of the microorganism; evidence of no impact on human and animal health and no harmful effects on the environment (toxicology and ecotoxicology); proof of the substance’s effectiveness for its intended purpose; the absence of prohibited residues; and a description of the formulation and production method (including stability studies, shelf life, storage, and quality control) [111,112,113].

In the safety analysis of yeasts, there is still no predefined safety assessment protocol with standardized methods [85,114]. According to the EFSA, for a microorganism to meet the criteria of the Qualified Presumption of Safety (QPS) system, genetic sequencing of the species must be performed to identify potential metabolic pathways related to toxigenicity or antifungal resistance, and this assessment must also be based on prior knowledge about the species [114,115]. For the FDA, a microorganism is considered GRAS only if its safety is evaluated at the strain level through sequencing [115]. Larini et al. [85] performed genetic sequencing and the genome analysis of M. pulcherrima strains NRRL Y-7111T, NRRL Y-48695, CBS 10357, and NRRL Y-48712, and stated that these strains can be considered safe due to the absence of pathogenic virulence factors, the low risk of antifungal resistance, the lack of chemicals such as biogenic amines, and precise taxonomic identification.

In-depth knowledge of the mechanisms of action of new microorganisms considered antagonists is important for developing new products [62]. Identifying and studying these new antagonists has been the focus of research in recent years, with current efforts aimed at finding those that can reduce the occurrence of postharvest diseases in fresh fruits [6,18,41,44,62]. However, studies on formulations and stability are also important for scaling up and regulating commercial bioproducts [19].

Buhlmann et al. [19] analyzed the stability of *M. pulcherrima* in liquid and dry formulations over time. They observed that dry formulations (10% SMP, 10% sucrose, and lyophilized) were more effective and maintained *M. pulcherrima* viability at 80% after 43 months when stored at 4 °C. Liquid formulations, with cells suspended in water, maintained 15.5% viable cells after 300 days at 22 °C, while still retaining biocontrol activity.

Variability between strains is another important factor in developing commercial products, as it can lead to significant differences in biological performance, including resistance to environmental stresses, colonization ability, and antagonistic activity. This variability, along with sensitivity to environmental factors such as temperature and humidity, contributes to the differences observed between laboratory studies and large-scale applications [22,52]. Additionally, compatibility with application matrices, such as waxes and biopolymers used in postharvest coatings, remains poorly studied, highlighting a significant gap in the development of these commercial products [35,44].

Currently, some products on the market use *M. pulcherrima* cells, although many were developed for winemaking. This shows that these yeasts can be used on an industrial scale. Generally, their function involves competing for space and nutrients, which prevents undesirable microorganisms from developing [44,59,76]. Although the commercial *M. pulcherrima* products mentioned were developed for oenological applications, their use demonstrates the proven biocontrol capacity of this species. The studies selected in this review reinforce the antagonistic properties of *M. pulcherrima* and its potential as a candidate for developing biofungicides that could replace synthetic fungicides.

## 4. Strengths and Limitations of the Scoping Review

This scoping review demonstrated both strengths and limitations. Notably, the use of a structured protocol and the PCC strategy, following JBI guidelines, ensured greater methodological transparency and reproducibility from the outset. The process included clearly defined steps for identifying, screening, and including articles, with independent evaluations and consensus decisions among reviewers. Additionally, adherence to the PRISMA-ScR protocol provided clarity in presenting results, aligning with the objectives of this review. The bibliometric analysis also identified patterns, trends, and thematic relationships regarding *M. pulcherrima*, organizing the evidence without replacing critical analysis.

Regarding the limitations, restricting the review to original articles from a specific period may have excluded relevant studies, particularly those published outside that timeframe. Including only studies in English may have introduced linguistic bias, limiting access to regional and gray literature, where additional information may have been lost. Additionally, variations in fruit storage conditions, inoculation methods, and treatment efficacy evaluation criteria make direct comparisons between studies difficult. The description of the results in this review also prevents robust quantitative conclusions about the efficacy of *M. pulcherrima*, as a larger number of studies in some countries may reflect greater scientific output rather than greater use of biocontrol.

In addition, heterogeneity among the pathogens evaluated, yeast formulations, and experimental protocols may influence variation in results. Therefore, the findings are presented as a mapping of evidence and application patterns, not as general estimates of efficacy.

## 5. Conclusions

This review highlights the promising role of the yeast *M. pulcherrima* as a biocontrol agent against postharvest phytopathogens. The studies analyzed, including both in vitro and in vivo studies, consistently demonstrate this yeast’s ability to inhibit the growth and infection caused by several economically significant fungi responsible for major losses, especially in fruits.

Despite the reported advances, most studies are still limited to laboratory experiments and small-scale trials. Developing commercial products based on *M. pulcherrima* offers a sustainable strategy for controlling postharvest diseases. Formulating biofungicides with this yeast provides a viable alternative to conventional chemical fungicides, whose residues can affect human health and the environment, while also minimizing issues with pathogen resistance.

*M. pulcherrima* should be considered an effective antagonistic organism, especially since investments in new research and the development of biofungicides are justified by its diverse mechanisms of action and proven effectiveness. The large-scale adoption of biocontrol strategies using this yeast depends on integrating established scientific knowledge and technological solutions that enable its commercial application.

## Figures and Tables

**Table 1:** Journals publishing research on *M. pulcherrima* as a postharvest biocontrol agent.

Journals	Number of Papers	References
International Journal of Food Microbiology	8	[16,26,28,37,40,41,42,43]
Food Microbiology	5	[9,14,15,44,45]
Biological Control	5	[20,21,27,46,47]
Microorganisms	5	[29,31,48,49,50]
Postharvest Biology and Technology	4	[4,35,51,52]
Toxins	2	[36,53]
Foods	2	[23,54]
Journal of the Science of Food and Agriculture	2	[55,56]
Biotechnology Research International	1	[57]
Journal of Phytopathology	1	[58]
The Journal of Horticultural Science and Biotechnology	1	[59]
Acta Horticulturae	1	[10]
BMC Microbiology	1	[60]
Food Science and Biotechnology	1	[24]
Phytopathology	1	[61]
Fermentation	1	[62]
Molecular Microbiology	1	[33]
Journal of Integrative Agriculture	1	[25]
Horticulturae	1	[19]
Agronomy	1	[30]
Food and Science Nutrition	1	[63]
Frontiers in Microbiology	1	[64]
Food Bioscience	1	[65]
Journal of Food Science	1	[13]
Food Energy Security	1	[5]
Horticultural Plant Journal	1	[66]
LWT—Food Science and Technology	1	[67]
Current Microbiology	1	[22]
Plant Physiology and Biochemistry	1	[68]
Journal of Fungi	1	[69]
Microbiology Research	1	[34]
International Journal of Agriculture and Biosciences	1	[70]
Current Trends in Natural Science	1	[71]
Applied Science	1	[72]
Molecules	1	[73]
Plant Omics Journal	1	[74]
Total	61	

**Table 2:** *M. pulcherrima* yeasts isolated from different materials and their respective culture media used for cell growth.

Sources	*M. pulcherrima* Strain	Cell Growth Medium	Cell Culture Conditions	Reference
Grape surface	DiSVA 267, 269, 467, 476, 489, 1069, 1067	YEPG liquid medium (yeast extract 10 g·L^−1^, peptone 10 g·L^−1^, and D-glucose 20 g·L^−1^)	25 °C/24 h-48 h	[16,50,52]
Vineyards of Türkiye	UMY15	YEPG liquid medium (yeast extract 10 g·L^−1^, peptone 20 g·L^−1^, and glucose 20 g·L^−1^)	30 °C/overnight at 130 rpm	[57]
Jujubes fruit surface	-	NYDB liquid medium (nutrient broth 8 g·L^−1^, yeast extract 5 g·L^−1^ and dextrose 10 g·L^−1^)	28 °C/24 h at 140–200 rpm	[13,58,59,74]
Minimally processed pomegranate and olive brine	MPR3	PD solid medium (pH 6.0 and 4.5); YEPD solid medium (yeast extract 10 g·L^−1^, peptone 10 g·L^−1^, dextrose 20 g·L^−1^ and agar 20 g·L^−1^); YNBS liquid medium (supplemented with 100 mM glucose) for the biofilm formation	25 °C/48–72 h	[10,14,15,44,45]
Different cultivars of figs (*Ficus carica* L.)	L672	NYDB liquid medium (nutrient broth 8 g·L^−1^, yeast extract 5 g·L^−1^ and dextrose 10 g·L^−1^)	25 °C/24 h at 120 rpm	[9,55]
Apple blossoms; apple phyllosphere	APC 1.2; wild-type isolate APC 1.2	PD solid medium; PD liquid medium	22 °C; 22 °C/overnight at 150 rpm	[19,33,40,60]
Malbec wine grapes	LP132.1	MYGP solid medium	28 °C/48–72 h	[26]
Wine and fruits	-	YEPG liquid medium (yeast extract 10 g·L^−1^, peptone 20 g·L^−1^ and glucose 20 g·L^−1^)	-	[62]
Mango orchard soil	-	PD liquid medium	28 °C/72 h at 110 rpm	[24]
Vineyard	P01A016 and P01C004	YEPD liquid medium (peptone 20 g·L^−1^, dextrose 20 g·L^−1^, yeast extract 10 g·L^−1^); YEPG solid medium; PD liquid medium	25 °C/48 h under a 12 h photoperiod; 28 °C/24 h at 250 rpm	[61,68]
Red grapes	RCM2	PD solid medium	22°C	[27]
Rhizosphere soil of a mango tree	-	PD liquid medium	28 °C/48 h	[25]
Table grapes	Mp8, Mp11, Mp22, Mp36, Mp43, Mp45, Mp46, Mp47 e Mp53	YEPD solid medium (yeast extract 10 g·L^−1^, peptone 20 g·L^−1^, dextrose 20 g·L^−1^, agar 20 g·L^−1^; pH 4.5)	24–48 h	[28]
Grapes	Mp-16, Mp-22, Mp-23, Mp-30, Mp-35, Mp-50	YME solid medium (malt extract 3 g·L^−1^, yeast extract 3 g·L^−1^, peptone 4 g·L^−1^, dextrose 10 g·L^−1^ and agar 20 g·L^−1^; pH 6.8); YEPD liquid medium (yeast extract 10 g·L^−1^, peptone 20 g·L^−1^ and dextrose 20 g·L^−1^; pH 6.8)	28 °C/48 h; 28 °C/48 h at 150 rpm	[20]
Grape juice	N20/006, Ale4 and Pr7	YEPD solid medium (yeast extract 10 g·L^−1^, peptone 20 g·L^−1^, dextrose 20 g·L^−1^, agar 20 g·L^−1^)	25 °C/48 h	[31]
Grape berries	H12.08, A05.01, B05.02, F12.06, G12.07, GP8, and E20671	YEPD liquid medium (pH 5.5)	25 °C/24 h	[29]
Apple skins	Y33, Y29, and Y24	YEG liquid medium (yeast extract 90 g·L^−1^ and glucose (D (+) glucose monohydrate 200 g·L^−1^)	30 °C/24 h	[36]
Sweet and sour cherries	PA-6-26.1 and PC-1-48.2	YEPD solid medium		[48]
Loquat leaves	E1	YED liquid medium; malt extract decoction	28 °C/24 h at 180 rpm; 28 °C/ 2–3 days at 150 rpm	[46,69]
Apple, raspberry and strawberry fruits; strawberry flowers	NCYC 747 e 2321; J2, J3, J6, TK1 e M4	ME solid medium; YPD liquid medium or different variants of acid whey-based supplemented medium	25 °C/24 h; 25 °C/72 h at 160 rpm	[30]
Peel surface of cider apples	-	YEG liquid medium	28 °C/overnight	[35]
-	T-2	YEPD liquid medium	28 °C/180 rpm	[63]
Pear leaves and fruits	V2 and V7	YED liquid medium	30 °C	[49]
Grapes (Vitis vinifera)	Mp-22 and Mp-30	YME solid medium; YNBS liquid medium (with additional glucose 20 g·L^−1^ and ammonium sulfate 5 g·L^−1^, pH 6.8); YPD liquid medium	28 °C/2 days at 150 rpm	[51]
	632	PD solid medium		[65]
Grapes, blackberries, *Amaranthus retrofexus*, corn tassel, bean leaves and hawthorn	*M. aff. pulcherrima* (26-BMD, 32-AMM, DN-HS, DN-MP e DN-UY)	ME solid medium supplemented with chloramphenicol 0.1 g·L^−1^	25 °C/72 h	[53]
Wine-related environments	-	YEPG solid medium (yeast extract 5 g·L^−1^, peptone 10 g·L^−1^, glucose 20 g·L^−1^ and agar 20 g·L^−1^)	28 °C	[64]
Grapes	Mp-30	YM solid medium or YEPD liquid medium	28 °C/48 h or 28 °C/48 h at 150 rpm	[21]
Hawthorn fruits	P01A016	YEPG solid medium (yeast extract 10 g·L^−1^, peptone 20 g·L^−1^, glucose 20 g·L^−1^ and agar 20 g·L^−1^)	25 °C/48–72 h	[47]
Table grapes	CICC 33433, CICC 1467, CICC 32343, CICC 33447, and CGMCC 2.3314	YEPD solid medium (peptone 20 g·L^−1^, dextrose 20 g·L^−1^, yeast extract 10 g·L^−1^, agar 20 g·L^−1^), enriched or not with FeCl_3_ 5 mg·L^−1^	28 °C/48 h	[23]
Amaranthus retrofexus, corn tassel and grape leaf	DN-HS, DN-MP and DN-UY	YEPD liquid medium	25 °C/48 h	[22]
Surface of blueberries	T-2	YEPD solid medium	28 °C/48 h	[66,67]
Table grapes (*Vitis vinifera* L.)	34-UEM	YEPG liquid medium (yeast extract 10 g·L^−1^, peptone 20 g·L^−1^, and glucose 20 g·L^−1^)	28 °C/24 h at 200 rpm	[5]
	Mp22, Mp36, Mp43	YEPG liquid medium (yeast extract 10 g·L^−1^, peptone 20 g·L^−1^, glucose 20 g·L^−1^)	25 °C/48 h	[4]
Grape Red Globe	RCM2 and ULA146	YEPG liquid medium (glucose 0.4 g·L^−1^, peptone 5 g·L^−1^, yeast extract 5 g·L^−1^)	4 °C/7 days	[37]
Grape berry	sa5	YEPD solid medium (yeast extract 10 g·L^−1^, peptone 20 g·L^−1^, D-dextrose 20 g·L^−1^ and agar 20 g·L^−1^)	28 °C	[71]
Grape peel	WM05	YEPD liquid medium	28 °C/20 h at 200 rpm	[41]
Grape and apple epidermis	MP01, MP02, MP06, MP07, MP08, MP11, MP14; *M. aff. pulcherrima* (MP03, MP04, MP05, MP09, MP10, MP12, MP13)	NYDB liquid medium	28 °C/2 days at 200 rpm	[42]
Table grapes	XX04	YM liquid medium	28 °C/20–24 h at 180 rpm	[43]
Apple and pear peel surface	MP-01, MP-02, MP-03, MP-04, MP-05, MP-06, MP-07 and MP-08	NYDA solid medium and SD solid medium	28 ± 2 °C/48 h	[70]
Apple, raspberry fruits and strawberry flowers	D1, D2, D3, D4 and TK1	YEPG liquid or solid medium; YEPG complex medium (yeast extract 5.0 g·L^−1^, soy peptone 5.0 g·L^−1^, and glucose 2.6 g·L^−1^)	25 °C/48 h at 150 rpm; 30 °C/48 h; 25 °C/48 h at 180 rpm (pH 5)	[72,73]
Wine	UMY1472	YEPG liquid medium (peptone 20 g·L^−1^, yeast extract 10 g·L^−1^, glucose 20 g·L^−1^)	24–48 h	[34]

YEPD (Yeast Extract Peptone Dextrose); YEPG (Yeast Extract Peptone Glucose); YEG (Yeast Extract Glucose); YED (Yeast Extract Dextrose); NYDB (Nutrient Yeast Dextrose Broth); NYDA (Nutrient Yeast Dextrose Agar); YM (Yeast Mannitol); ME (Malt Extract); YME (Yeast Malt Extract); YNBS (Yeast Nitrogen Base); PD (Potato Dextrose); YPD (Yeast Potato Dextrose); and SD (Sabouraud Dextrose).

**Table 3:** Use of *M. pulcherrima* yeast as a postharvest biocontrol agent.

Host Fruit and Vegetables	Target Pathogens	Treatment Combination with *M. pulcherrima*	Conclusions about *M. pulcherrima* Modes of Action	Main Results (*In Vivo* Test)	Reference
Sweet cherries	*M. fructicola*		Colonization and persistence on the surface of fruits.	The application of a suspension of *M. pulcherrima* (10^7^ cells·mL^−1^) reduced the infection rate in cherries by 62% compared to the control.	[52]
Apple slices and grape juice	*Penicillium roqueforti*, *P. italicum*, *P. expansum*, *Fusarium* sp., *Rhizopus* sp., *Aspergillus niger*, *Aspergillus oryzae*, and *Aspergillus parasiticus*		Competition for iron ions.	*M. pulcherrima* was 100% effective in the biocontrol of *P. roqueforti*, *Fusarium* sp., and *A. oryzae*.	[57]
Jujube fruit	*A. alternata*	UV-C (5 kJ·m^2^ for 15 min.)		The combination of *M. pulcherrima* and UV-C reduced the incidence of *alternaria* rot by 44% in artificially inoculated fruits.	[13]
Table grapes	*B. cinerea*		VOCs’ production, competition for iron ions, and biofilm formation.	*M. pulcherrima* MPR3 controlled gray mold in grape berries.	[14]
Jujube fruit	*P. citrinum* and *A. alternata*	2% (*p*/*v*) of CaP	Competition for nutrients and space.	The combination of 2% (*w*/*v*) CaP and 10^8^ cells. mL^−1^ of the yeast *M. pulcherrima* reduced disease incidence by 27.3% for *P. citrinum* and 31.4% for *A. alternata* in artificially inoculated fruits.	[59]
Jujube fruit	*P. citrinum*	Microwave for 2 min (2450 MHz)		The combination of microwave treatment and immersion in *M. pulcherrima* solution (10^8^ cells·mL^−1^ for 1 min) reduced natural disease incidence by 21.67% after storage at 2 ± 1 °C for 45 days and at 22 °C for 7 days.	[74]
Jujube fruit	*P. citrinum*	Microwave for 2 min		The percentage of deteriorated fruit treated with the combination of microwaves and *M. pulcherrima* was only 6.2%, compared to 28.6% in the control group.	[58]
Grapes and Mandarin	*B. cinerea*, *P. digitatum*, and *P. italicum*	LBG bioactive coating (0.5% and 1.0%)	Induction of resistance, indicated by increased peroxidase and superoxide dismutase activity	LBG coating at 1.0% incorporated with *M. pulcherrima* reduced the incidence and severity of the disease in artificially inoculated mandarin fruits.	[44]
Apples and nectarines	*B. cinerea* CECT20518, *M. laxa* CA1, *P. expansum* M639, and *C. cladosporioides* M310 and M624			*M. pulcherrima* L672 was most effective in inhibiting *P. expansum* (56% in apples; 69.4% in nectarines), *B. cinerea* (100% in apples; 57% in nectarines), and *M. laxa* (41.7% in apples; 54.5% in nectarines). The percentage inhibition of *C. cladosporioides* in nectarines was 52.5% for M310 and 56.57% for M624.	[9]
Strawberries and tangerines	*P. digitatum, P. italicum* and *B. cinerea*		VOCs’ production	VOCs produced by *M. pulcherrima* immobilized in hydrogel spheres reduced the severity of the disease and the lesion diameter caused by *P. digitatum* in mandarins and *B. cinerea* in strawberries.	[45]
Sweet cherries	*P. expansum* M639	MAP		Packaging with MAP (microperforated with three holes) combined with *M. pulcherrima* controlled the development of *P. expansum* in wounded cherries for up to 21 days in refrigerated storage.	[40]
Strawberries	*M. fructicola*, *A. alternata*, *Aspergillus carbonarius*, *P. digitatum*, *Cladosporium* spp., and *Colletotrichum* spp.		VOCs’ production	Strawberries exposed for 48 h to VOCs produced by 6-day liquid cultures of *M. pulcherrima* showed a 40% reduction in the McKinney Index for gray mold.	[16]
Mango	*C. gloeosporioides*		Competition for nutrients and space.	Treatment with *M. pulcherrima* inhibited changes in total soluble solids content, total acidity, and vitamin C in mango fruits compared to untreated fruits.	[24]
Grapes	*A. alternata*			The *M. pulcherrima* strains LP122.2, LP128.2, and LP131.2 completely prevented *A. alternata* infection in grape berries, achieving an infection rate of 0% when applied 2 h before pathogen inoculation.	[26]
Grapes	*B. cinerea* 111bb, 207a, 207cb and 407cb			*M. pulcherrima* P01A016 suppressed the growth of all *Botrytis* isolates in inoculated grape berries.	[61]
Grapes	*A. alternata*			After application of *M. pulcherrima* inoculum (10^6^ cells·mL^−1^), the incidence of *A. alternata* was lower on the surface of the wounded berries; however, the disease incidence was higher than in the SO_2_ treatments.	[27]
Mango	*C. gloeosporioides*	SA solution (50 mg·L^−1^) and CaCl_2_ solution (1 g·L^−1^)		The combination of yeast, SA, and CaCl_2_ prevented anthracnose development in the fruit and delayed ripening by 24 days.	[25]
Table grapes	*P. expansum* PSS4, PSS6, PRG2, and PRG3			Three different isolates of *M. pulcherrima* (Mp22, Mp36, and Mp43) reduced the incidence and severity of blue mold in artificially inoculated fruits.	[28]
Apples	*Neofabraea vagabunda*			*M. pulcherrima* reduced the diameter of lesions caused by *N. vagabunda* by more than 50% in artificially inoculated apple fruits.	[19]
Cherry tomatoes and grapes	*B. cinerea*			The Mp-22 and Mp-30 strains of *M. pulcherrima* almost completely inhibited gray mold symptoms in tomatoes, reducing disease severity by 97% during the 14-day storage period. These strains also provided complete protection against the disease in grapes.	[20]
Grapes	*B. cinerea*		Production of cell wall-degrading enzymes.	The application of *M. pulcherrima* (10^4^ cells·mL^−1^) reduced the severity of the disease caused by *B. cinerea* by 95.3% compared to the control.	[31]
Grapes	*A. carbonarius*			The application of *M. pulcherrima* GP8 to grape berries inhibited the growth of *A. carbonarius*, reducing the growth diameter by 76% compared to the control.	[29]
Apples	*P. expansum*			Applying a spore suspension of *M. pulcherrima* Y29 to the surface of apples inhibited the development of blue mold for up to 10 days of storage at 21 °C.	[36]
Loquat	*Pestalotiopsis vismiae*		Competition for space and nutrients, biofilm formation, VOCs’ production, and induction of resistance in fruits.	*M. pulcherrima* E1 (10^9^ cells·mL^−1^) reduced rot incidence in loquats by 31.81% compared to the control treatment.	[46]
Grapes	*B. cinerea*			The *M. pulcherrima* DiSva 269 strain was effective in controlling *B. cinerea*, showing lower disease severity than the commercial formulation with *A. pullulans* used as a positive control.	[50]
Tomatoes, apples and grapes	*B. cinerea*		Metabolites produced by *M. pulcherrima*.	Three metabolites produced by *M. pulcherrima* (3-amino-5-methylhexanoic acid, biphenyl-2,3-diol, and sinapaldehyde) at a concentration of 100 mM were effective in controlling *B. cinerea* infection in tomatoes, apples, and grapes, reducing both the incidence and severity of the disease.	[51]
Pistachio nuts	*Aspergillus flavus*		Production of extracellular enzymes and VOCs.	*M. pulcherrima* inhibited the growth and sporulation of *A. flavus* in wounded pistachio seeds.	[15]
Potatoes	*F. oxysporum*, *F. sambucinum*, *R. solani*, *A. solani*, *A. tenuissima*, *A. alternative*, *C. coccodes*, *P. exigua*, *P. carotovorum*, and *S. scabiei*		Iron depletion, enzymatic activity, and organic acid production.	*M. pulcherrima* TK1 completely or partially inhibited fungal development (100–30%) and achieved 40% inhibition against *S. scabiei*. It was not able to inhibit the symptoms of the disease caused by *P. carotovorum* in artificially inoculated potato seeds.	[30]
Apples	*P. expansum*	Edible coatings made from a matrix of apple pomace waste.		The coating containing *M. pulcherrima* delayed the development of blue mold in artificially inoculated fruits stored at 21 °C for 17 days.	[35]
Sweet cherries		MAP		The combination of MAP with *M. pulcherrima* yeast improved control of microbiological deterioration, similarly to the fungicide treatment, and reduced fruit weight loss during storage.	[55]
Apples	*B. cinerea* and *Erysiphe necator*		Resistance induction.	The *M. pulcherrima* (Mp-30) strain limited the development of *B. cinerea* infection in apple fruits when inoculated with 10^4^ or more yeast cells per wound.	[21]
Mandarins	*P. digitatum*	Combination of the yeasts *Meyerozyma guilliermondii*, *Hanseniaspora uvarum*, and *M. pulcherrima*	Biofilm formation and competition for nutrients.	The combined use of yeasts produced a synergistic effect, increasing the effectiveness of biocontrol.	[47]
Apples	*P. expansum*		Production of VOCs, biofilm formation, and inhibition of spore germination	Symptoms of disease caused by *P. expansum* in wounds on artificially inoculated apples were reduced.	[22]
Grapes	*B. cinerea*			*M. pulcherrima* 34-UEM cells were effective in biocontrol against *B. cinerea*, significantly reducing disease incidence by 89.4% and lesion diameter by 88.7% in grape berries.	[5]
Table grapes	*P. expansum PSS6*	NaHCO_3_ (0.3% *w*/*v*) + *M.pulcherrima* Mp22 and Mp36		The combination of NaHCO_3_ and *M. pulcherrima* reduced the symptoms of blue mold in grapes stored for 30 days at 2 °C.	[4]
Grapes	*A. alternata* AU133 and AU159	Bioactive extracts from vine shoots and stems + CHI		The combination of *M. pulcherrima* with shoot extracts, stem extract, and CHI did not increase the effectiveness of the yeasts against the pathogen; however, *M. pulcherrima* ULA146 alone controlled the size and diameter of the lesions, similarly to the commercial treatment.	[37]
Blueberries	*B. cinerea*		VOCs’ production	VOCs emitted by *M. pulcherrima* T-2 reduced rot caused by *B. cinerea* in blueberry fruits. The total flavonoid content in the fruits was higher after treatment with VOCs, which was associated with increased resistance to the pathogen.	[67]
Blueberries	*B. cinerea*		VOCs’ production	*M. pulcherrima* T-2 reduced rot symptoms caused by *B. cinerea*, decreased fruit mass loss, and increased total soluble solids (TSS), total saturated fat (TA), and vitamin C levels in blueberry fruits stored at 25 °C and 85% RH.	[66]
Mandarins	*Geotrichum citri-aurantii*; *P. digitatum* and *P. italicum*		Nutrient and space competition, surface colonization, biofilm formation, VOCs’ production, resistance induction, and iron competition	Treatment with *M. pulcherrima* (10^8^ cells·mL^−1^) completely inhibited the development of green and blue mold in the fruit and reduced the appearance of sour rot symptoms by 98% in artificially inoculated fruit. It also reduced naturally occurring rot symptoms by 64.8% over 180 days of storage.	[41]
Apples	*B. cinerea, A. alternata*, *A. tenuissima*, *C. coccodes*, *Fusarium oxysporum*, *F. sambucinum*, *M. laxa*, *P. exigua* and *Venturia inaequalis*.			Fewer than 50% of the fruits treated with a suspension (10^8^ cells·mL^−1^) of *M. pulcherrima* D2 showed symptoms of diseases caused by *B. cinerea*, *A. alternata,* and *A. tenuissima* for up to 31 days of storage at 21 ± 2 °C.	[72]
Grapes	*B. cinerea*	Phytohormone 24-epibrassinolide (0.8 mg·L^−1^)		The synergistic effect of the phytohormone combined with a solution of *M. pulcherrima* (10^9^ cells·mL^−1^) controlled rot and maintained fruit firmness for 60 days, increased the content of phenolic compounds, flavonoids, and anthocyanins, reduced oxidative stress, and extended the fruit’s shelf life.	[68]
Apple	*P. expansum*			The use of an MP-03 yeast solution (10^8^ cells·mL^−1^) on artificially inoculated fruits reduced *P. expansum* infection by up to 65%. Furthermore, applying this solution to healthy fruits allowed apples to be stored for up to 120 days at 1–2 °C, maintaining approximately 74% healthy fruit.	[70]
Loquats	*P. vismiae*			The application of *M. pulcherrima* E1 (10^8^ cells·mL^−1^) to artificially inoculated fruits inhibited the development of *P. vismae* in fruits stored at room temperature by approximately 77%.	[69]
Grapes	*B. cinerea*		Colonization capacity, space competition, and VOC activity.	The use of an MP07 yeast solution (10^8^ cells mL^−1^) on artificially inoculated fruits inhibited *B. cinerea* infection by approximately 80%. The VOCs produced by MP14 inhibited almost 100% of *B. cinerea* growth in fruits stored at 25 °C and 85% relative humidity.	[42]

CaP (calcium propionate); UV-C (ultraviolet-C light); LBG (locust bean gum); MAP (modified atmosphere packaging); SA (salicylic acid); CaCl_2_ (calcium chloride); CHI (chitosan); VOCs (volatile organic compounds); TSS (total soluble solids); and TA (total acidity).

**Table 4:** VOCs produced by *M. pulcherrima* and their biological activities.

Molecule	Structure	Individual/Synergistic Activity	Reference
Ethyl acetate	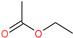	Individual antifungal properties against *B. cinerea*	[16,46]
Isoamyl alcohol or 1-butanol, 3-methyl-	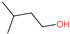	Individually inhibits the mycelial growth	[16,29,46]
Isoamyl acetate or 1-butanol, 3-methyl acetate	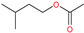	Individual antifungal properties against *B. cinerea*	[16,46,67]
Phenylethyl alcohol or 2-phenyl-ethanol	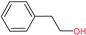	Individually inhibits *B. cinerea* and *Pestalotiopsis vismiae* mycelial growth and spore germination	[16,29,46,67]
Acetic acid	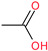	Individual and synergistic fungal growth and spore germination reduction	[29,67]
Isobutyl alcohol or isobutanol	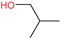	Synergistic fungal growth inhibition	[16,29]
n-caprylic acid or octanoic acid	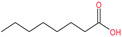	Individual fungal growth and spore germination reduction	[29,67]
1-hexanol	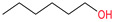	Individual *P. vismiae* growth reduction	[46]
2-ethyl-1-hexanol	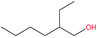	Individual fungal growth reduction	[67]
Benzaldehyde	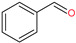	Individual fungal growth reduction	[67]
Benzyl alcohol	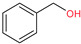	Individual fungal growth reduction	[67]
3-hydroxy-2-butanone	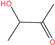	Individual fungal growth reduction	[67]
2,5-dimethyl-pyrazine	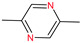	Individual *B. cinerea* growth reduction	[67]
Amyl alcohol	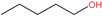	Synergistic fungal growth inhibition	[16]

The chemical structures were drawn ACD/ChemSketch v. 2025.2.0.

## Data Availability

The data that support the findings of this study are available from the corresponding author upon reasonable request.

## Supplementary Materials

The following supporting information can be downloaded at: https://doi.org/10.17605/OSF.IO/3HCRS (accessed on 6 April 2026). Table S1: Preferred Reporting Items for Systematic reviews and Meta-Analyses extension for Scoping Reviews (PRISMA-ScR) checklist. Table S2: Search strategies. Table S3: List of included and excluded studies with exclusion criteria.
